# Quantitative Evidence to Challenge the Traditional Model in Heterogeneous Catalysis: Kinetic Modeling for Ethane Dehydrogenation over Fe/SAPO-34

**DOI:** 10.1021/jacsau.2c00576

**Published:** 2022-12-19

**Authors:** Peng Chen, Ying Liu, Yarong Xu, Chenxi Guo, P. Hu

**Affiliations:** † Key Laboratory for Advanced Materials, Centre for Computational Chemistry and Research Institute of Industrial Catalysis, East China University of Science and Technology, Shanghai200237, China; ‡ Research Institute of Urumqi Petrochina Chemical Company, Urumqi83000, China; § Department of 5T Technology, Zhejiang SUPCON Technology Co., Ltd., Hangzhou310053, China; ∥ School of Chemistry and Chemical Engineering, The Queen’s University of Belfast, BelfastBT9 5AG, United Kingdom

**Keywords:** ethane dehydrogenation, density functional theory, zeolite, adsorbate−adsorbate interactions, microkinetic modeling

## Abstract

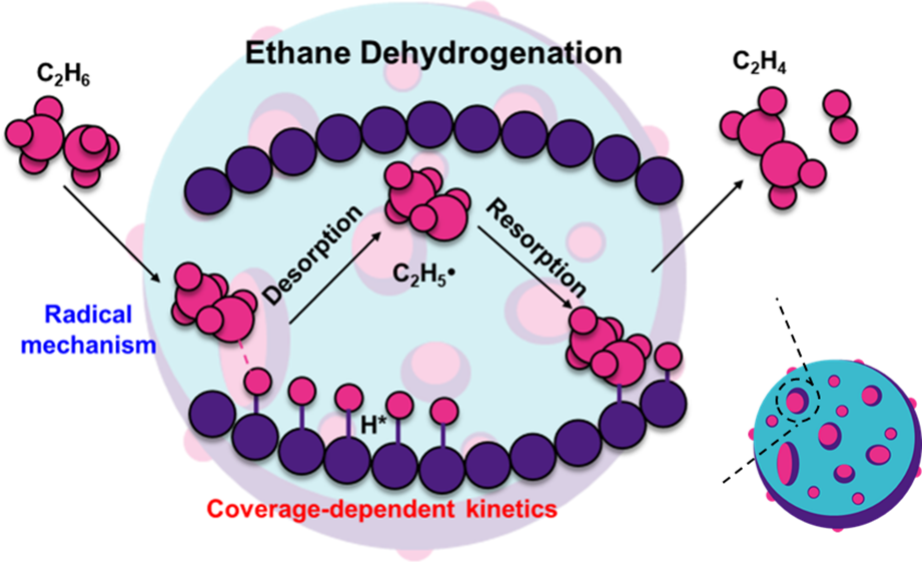

The production of ethylene from ethane dehydrogenation (EDH) is of great importance in the chemical industry, where zeolites are reported to be promising catalysts and kinetic simulations using the energetics from quantum mechanical calculations might provide an effective approach to speed up the development. However, the kinetic simulations with rigorous considerations of the zeolite environment are not yet advanced. In this work, EDH over Fe/SAPO-34 is investigated using quantum mechanical calculations with kinetic simulations. We show that an excellent agreement between the reaction rates from the self-consistent kinetic simulations using the coverage-dependent kinetic model developed in this work and the experimental ones can be achieved. We demonstrate that the adsorbate–adsorbate interactions are of paramount importance to the accuracy of kinetic calculations for zeolite catalysts. Our self-consistent kinetic calculations illustrate that the CH_3_CH_2_• radical rather than CH_3_CH_2_* is a favored intermediate. Perhaps more importantly, we reveal that the traditional model to describe catalytic reactions in heterogeneous catalysis cannot be used for the kinetics of the system and it may not be appropriate for many real catalytic systems. This work not only builds a framework for accurate kinetic simulations in zeolites, but also emphasizes an important concept beyond the traditional model.

## Introduction

Ethylene is an important building block to produce fibers, plastics, and other chemical intermediates.^1,2^ Previously, the steam cracking of hydrocarbons from naphtha is applied as a conventional way for the production of ethylene, which is far from satisfying the ever-increasing demand for energy and resources.^1,3^ Recently, shale gas has been regarded as a promising source for ethane. Accordingly, oxidative dehydrogenation (ODH) of ethane has emerged as a feasible route for ethylene production due to its exothermic feature and hindrance of coke formation.^4,5^ However, the overoxidation of ethylene results in CO_2_ and CO as byproducts, which makes the technology inefficient.^1,6^ On the other hand, nonoxidative ethane dehydrogenation (EDH) has substantial advantages in avoiding overoxidation issues. Nevertheless, it still suffers from a high energy requirement and low conversion due to the thermodynamic equilibrium and rapidly coking-induced deactivation of the catalysts.^1,7^ Solutions have been focused on developing highly selective and anticoking catalysts.^8,9^

Metal and metal oxide catalysts, such as Pt- and CrO_*x*_-based catalysts, have been reported to be interesting materials for nonoxidative dehydrogenation of propane,^1,10^ as well as other oxides containing V, Ga, Zn, and Mo.^1,11,12^ High activity and selectivity of ethylene from EDH were found on Pt-based catalysts, promoted by adding Zn,^13^ Sn,^14^ Ga,^15^ and In,^3^ where the issues of hydrogenolysis, isomerization reactions, and metal sintering have been investigated in the systems. Moreover, single-site/atom catalysts showed excellent performance for EDH, while the stability at high temperatures remains a tough problem. For example, Qiao et al. reported that the coordinated oxygen in Au/FeOx catalyst is rapidly dissolved in the reductive environment, even promoted with the increase of temperature.^16^ Although the industrial process of propylene from propane dehydrogenation has been well developed, the low intrinsic conversion (around 10%) and inferior stability (over 60% of the initial activity will be lost after few hours) caused by coke formation and sintering of particles^8,17^ make the process of ethylene production from EDH remain to be a great challenge. Interestingly, zeolites were found to be very promising, achieving outstanding stability with high-temperature resistance.^18,19^ Therefore, it becomes a feasible strategy to dope metals into the framework of zeolites, where the isolated metal atoms in zeolites display a high stability due to the strong anchoring effect of the framework.^7,20^ Meanwhile, different doping elements results in a variety of reactivities, which provides an opportunity toward catalyst design. For instance, a superior coke resistance with excellent activity for EDH was reported on the Fe-containing MFI siliceous zeolite in the presence of ethylenediaminetetraacetic sodium (FeS-1-EDTA).^21^

Currently, the design of a single-site/atom catalyst within the framework of zeolite was conducted experimentally following a scheme of “trial-and-error”. Investigations of EDH over zeolites using density functional theory (DFT) calculations not only are a unique approach to achieve an understanding of reaction details at the atomic level but also may be an efficient tactic to overcome the design problem. However, the complex mechanism with difficulties of kinetic calculations results in it still being a challenge in the field of theoretical catalysis: The catalytic process through the adsorbed ethyl group (CH_3_CH_2_*) in EDH was usually reported,^21,22^ while the free radical mechanism was also found to be a feasible path in the dissociation of C–H.^23,24^ It is of great importance to consider the competition between different pathways, which was confirmed to be vital toward a more accurate prediction of activity.^25^ But it is not clear how to treat the radical kinetically.

In addition, the surface coverage has been found to play a significant role in kinetic calculations.^26,27^ The coverage effect from adsorbate–adsorbate interactions was considered in some previous works, displaying a more accurate description of catalytic activity than those without the coverage effects when compared with experimental ones. Although some attempts have been made to perform kinetic simulations of alkane dehydrogenation reactions,^28−30^ thorough studies with consideration of the influence of the coverage effect on the reaction in zeolite systems have not yet been reported, to the best of our knowledge. It is clear, therefore, that the following issues still need to be addressed theoretically for EDH: (i) What is the favored reaction mechanism? (ii) Is the coverage effect important in zeolite systems? If the answer is yes, how can we carry out a self-consistent microkinetic modeling with the coverage effect for zeolite systems? We aim to answer these questions in our work.

Furthermore, another important issue we focus on in this work is a major drawback of the traditional model to describe the catalytic reactions in heterogeneous catalysis. The traditional model, namely, the open surface model, by which any catalytic cycle is depicted in three parts: (i) adsorption of reactants; (ii) surface reactions; and (iii) desorption of products. The surface reactions exclusively occur on the surface because of the nature of the open surface. This model is arguably the most fundamental model in heterogeneous catalysis and has been used everywhere, such as all the textbooks, reviews and papers in the literature. Can we use this model to describe the kinetics of our systems? Or more general, can one apply this model to the kinetics of any catalytic processes in heterogeneous catalysis? In this work, we also attempt to address these questions.

In this work, taking Fe/SAPO-34 and SAPO-34 as models, competitive reaction mechanisms for ethane to ethylene, namely the two pathways through CH_3_CH_2_* and CH_3_CH_2_•, respectively, were studied. The adsorbed H* is found to be the main adsorbate on acid sites in zeolites from our calculations. The coverage effects on both adsorption energies and activation barriers were calculated and found to be important, and an approach is developed, which allows us to carry out the coverage-dependent microkinetic modeling self-consistently.^31,32^ The obtained rate is compared to experimental results, which reveals the high reliability of the theoretical results. More importantly, we demonstrate that the traditional model to describe the catalytic reactions in heterogeneous catalysis cannot be used in our current system, which may be inapplicable to many real systems.

## Methods

### Computational Details

All the calculations in this work were based on spin-polarized DFT through the Vienna *Ab initio* Simulation Package (VASP).^33,34^ The DFT-D3 correction^35^ was employed to describe the effect of the van der Waals (vdW) interactions, which is of necessity in the investigation of zeolites with porous structures. The electron–ion interactions were described by the projector augmented wave (PAW) method proposed by Blöchl and implemented by Kresse.^36,37^ The transition states (TS) were determined by the scheme of constrained optimization,^38,39^ where some of them were further confirmed by the method of climbing image nudged elastic band (CI-NEB). The Perdew–Burke–Ernzerhof (PBE) functional was used as an exchange-correlation functional approximation,^40^ and a plane wave basis set with an energy cutoff of 450 eV was used. All the atoms were relaxed during either the structure optimization or the calculations for the transition states with the force convergency lower than 0.05 eV/Å.

### Structures

**Figure 1:**
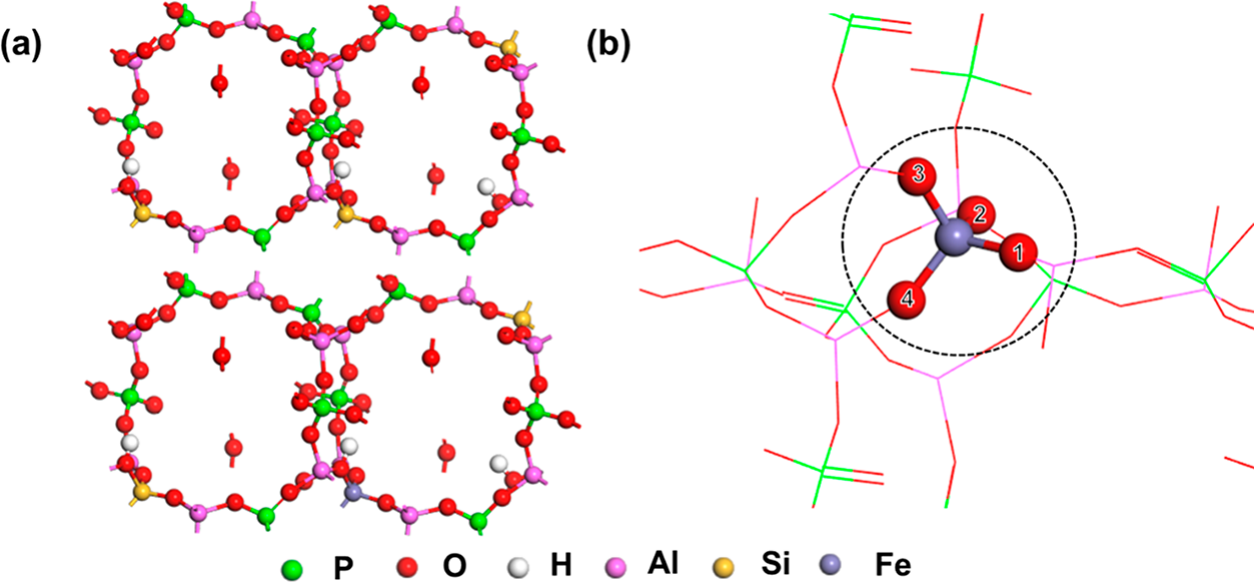
Structural illustrations of (a) SAPO-34 and Fe/SAPO-34 supercell and (b) active sites of Fe/SAPO-34. Atoms 1–4 show four adsorption sites around the central atom of Fe (Fe/SAPO-34) or Si (pristine SAPO-34).

The zeolite structures of Fe/SAPO-34 were modeled with a p (1 × 1 × 2) unit cell (Figure 1a), where the Monkhorst–Pack k-point of 1 × 1 × 1 with the gamma-centered scheme was used for the Brillouin zone integration due to the large size of the studied structures. As we mentioned in the Introduction, SAPO-34 is a specific type of silicoaluminophosphate zeolite with a chabazite (CHA) structure,^41^ where the composition of the unit cell is H_*x*_Si_*x*_Al_6_P_6–*x*_O_24_. The value of *x* can be 0, 1, or 2, corresponding to different Si/Al ratios. In this work, we built a SAPO-34 p (1 × 1 × 2) supercell with an *x* value of 1.5 to represent the zeolite,^42^ replacing the Si atoms in the CHA framework with Fe atoms, which is the Fe/SAPO-34 model, and the final chemical formula of the supercell is FeH_2_Si_3_Al_12_P_9_O_48_. There are four acid sites (i.e., the first neighbor oxygen atoms) around Fe over Fe/SAPO-34 or Si over SAPO-34, all of which were found to be important for the kinetics of systems (see more details in the section of Adsorbate–Adsorbate Interaction). Thus, they were included as the active sites for the dehydrogenation (see Figure 1b, labeled as 1, 2, 3, and 4), which was also reported in the previous work.^21^

### Adsorption Energies and Free Energy Corrections

The adsorption energy (*E*_ad_) is defined as
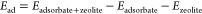
1where *E*_zeolite_, *E*_adsorbate_, and *E*_adsorbate+zeolite_ are the energies of the zeolite, adsorbate in the gas phase,^42^ and adsorbate in zeolites (including zeolite), respectively.

The free energies for adsorbates were corrected by the following equation:
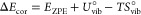
2where Δ*E*_cor_ refers to the free energy correction, *E*_ZPE_ refers to the correction of zero-point energy, *U*_vib_^°^ describes the inner energy correction from temperature and pressure, and *TS*_vib_^°^ represents the correction of entropy. Only vibrational motions were considered for adsorbates, and the corrected value Δ*E*_cor_ (Table S12, Table S13) was calculated using the VASPKIT code.^43^ Translational, rotational, and vibrational motions were all considered for the gas phase corrections, which were calculated by Gaussian code. Therefore, the standard Gibbs free energies (*G*°) are obtained by
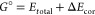
3where *E*_total_ refers to the total energy obtained from DFT calculations (see more details in Note 1 of the Supporting Information (SI)).

### Adsorbate–Adsorbate Interaction

In the system of Fe/SAPO-34, there are four oxygen atoms (acid sites) around the Fe atom adopted in the framework of the zeolite. A previous study reported that all the four individual acid sites can be adsorption sites of H.^21^ This suggests that some of these sites may be occupied simultaneously at the steady state during the catalytic process, and it may change the adsorption energies and the activation barriers in the system. Therefore, the coverage effect, i.e., the adsorbate–adsorbate interaction, must be included in order to obtain accurate kinetic results. To perform the self-consistent microkinetic calculations, continuous changes of coverages were used as an approximation in the model for simplicity (i.e., 0, 0.25, 0.5, 0.75, and 1 ML refer to the surface adsorption of 0, 1, 2, 3, and 4 H atoms on the acid sites, respectively, see Figure 1b).

The differential adsorption energy for adsorbed H* were calculated as follows:
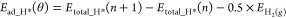
4
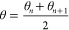
5where θ represents the relevant coverage on the surface and *n* is the number of H* (X* is defined as the adsorbed X). It is noted that the surface coverage for relevant differential adsorption energies was calculated as the center of coverage change (eq 5). The cross-interactions (CH_3_CH_2_*/H_sur_), namely, the coverage effect on adsorbed CH_3_CH_2_* from different numbers of H*, were calculated as
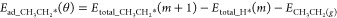
6
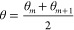
7where *m* is the total number of H* and CH_3_CH_2_*. The activation barrier of each elementary reaction (R1, R3, R4, R5) was obtained by subtracting the energy of the initial state from the energy of the transition state, and the coverage on the surface of each elementary reaction were calculated as
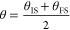
8where θ is the surface coverage, and θ_IS_ and θ_FS_ represent the coverages of the initial and final states, respectively.

The differential adsorption energy of different adsorbates (H* and CH_3_CH_2_*) and the reaction barrier of elementary step (R1, R3, R4, R5) under a certain coverage were calculated as
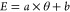
9where *E* represents the differential adsorption energy or the reaction barrier, θ is the coverage on the surface, and *a* and *b* represent the slope and intercept of the linear lines, respectively (Table S4).

The coverage-dependent reaction energies (Δ*G*_1_ - Δ*G*_5_) for elementary steps (R1 - R5 in Table 1) were calculated as follows:
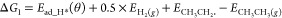
10
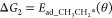
11
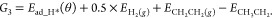
12
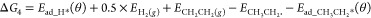
13
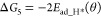
14where the coverage-dependent adsorption energies and reaction barriers were obtained from the two-line model in eq 9. Similar calculations were carried out for pristine SAPO-34, in which four oxygen atoms around a Si atom were taken into account in kinetic simulations (also see the section of Structures and Figure 1b) for comparison.

**Table 1:** Elementary Steps of EDH on the Clean Surface

no.	elementary steps^a^
R1	CH_3_CH_3_(g) + * → CH_3_CH_2_• + H*
R2	CH_3_CH_2_• + * → CH_3_CH_2_*
R3	CH_3_CH_2_• + * → CH_2_CH_2_(g) + H*
R4	CH_3_CH_2_* → CH_2_CH_2_(g) + H*
R5	2H* → H_2_(g) + 2*

a * represents an active site, and • represents a radical.

## Results and Discussion

### Reaction Mechanisms of EDH over Fe/SAPO-34 and SAPO-34 at Low Coverage

**Figure 2:**
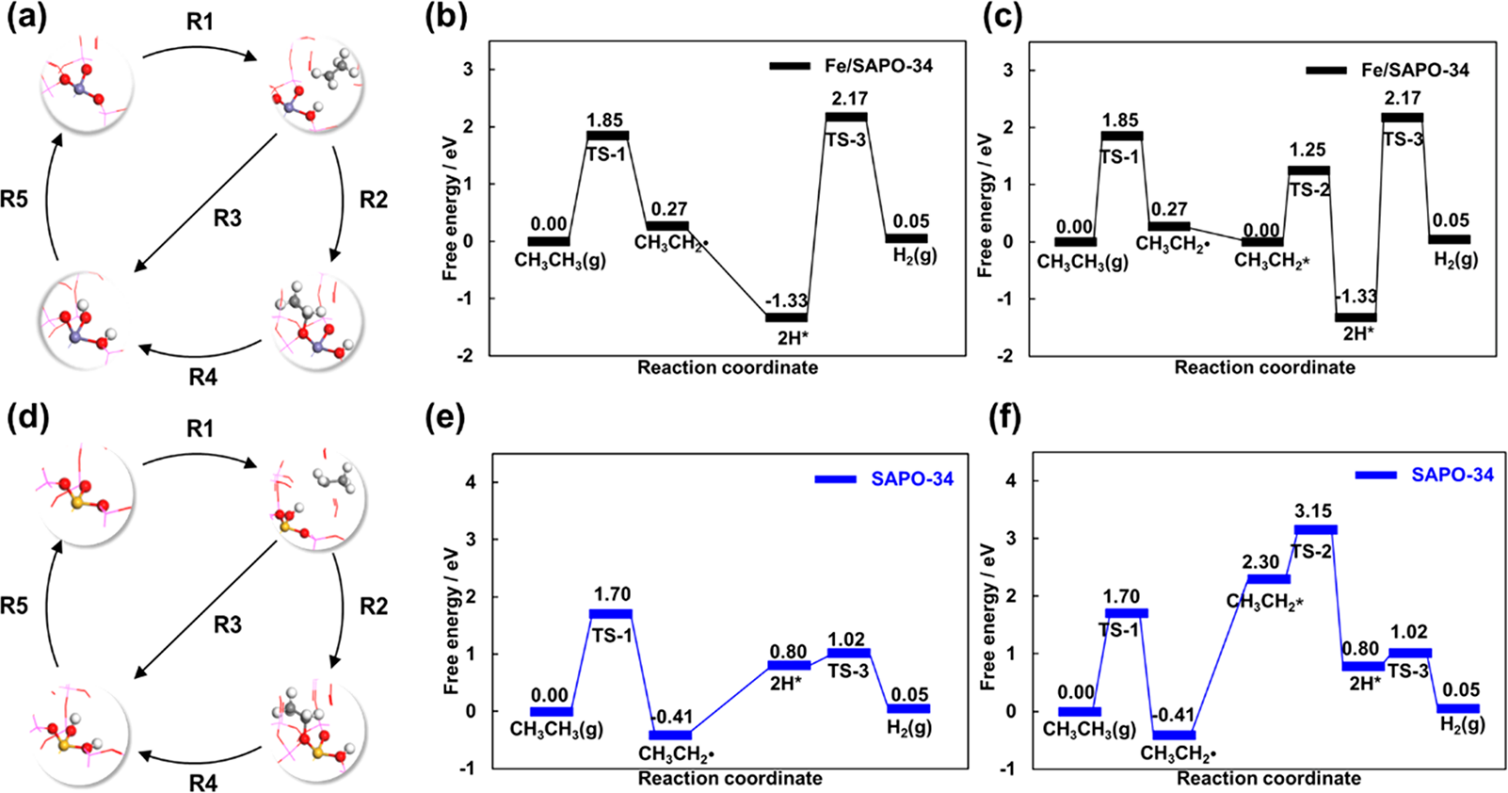
(a–f) Reaction mechanism of two pathways for ethane dehydrogenation (EDH) on the clean surface: CH_3_CH_2_• pathway (R1 + R3 + R5) and CH_3_CH_2_* pathway (R1 + R2 + R4 + R5) for (a) Fe/SAPO-34 and (d) SAPO-34. The free energy diagrams of EDH based on the CH_3_CH_2_• pathway over (b) Fe/SAPO-34 and (e) SAPO-34. The free energy diagrams based on the CH_3_CH_2_* pathway for (c) Fe/SAPO-34 and (f) SAPO-34. All the energies are corrected to the free energies at the temperature of 873 K (see eq 3). The reaction barriers (G_a_) and reaction free energies (Δ*G*) are listed in Table S2.

We started our investigation on the EDH mechanism over Fe/SAPO-34 and SAPO-34 using DFT calculations. Five elementary steps were considered in the EDH process (Table 1, R1–R5), and the clean surface was considered as the initial state in the catalytic cycle. The whole reaction cycle is displayed in Figure 2a for Fe/SAPO-34 and in Figure 2d for SAPO-34, respectively. A direct dissociation of CH_3_CH_3_ from the gas phase was studied due to the weak binding of CH_3_CH_3_ on the active site (Figure S1, Table S1). It is worth noting that CH_3_CH_2_• was considered as an intermediate after the first dehydrogenation of ethane (Table 1, R1) due to the fact that the CH_3_CH_2_• is formed after the transition state of the first dehydrogenation of ethane. Thus, the reaction pathway through free radical (CH_3_CH_2_•)^23,24^ was included and the adsorption and desorption of CH_3_CH_2_• (Table 1, R2) were taken into account in this work because the second dehydrogenation of CH_3_CH_2_ species can occur from either CH_3_CH_2_• (Table 1, R3) or CH_3_CH_2_* (Table 1, R4). Finally, the adsorbed H* is consumed by a direct H*-H* coupling (Table 1, R5). As a result, two pathways can be obtained for the EHD process, namely the CH_3_CH_2_• pathway (R1 + R3 + R5) and the CH_3_CH_2_* pathway (R1 + R2 + R4 + R5).

For these competitive pathways, the more favored reaction mechanism can be initially determined based on the barriers. For example, the rate-limiting step for EDH over Fe/SAPO-34 is found to be the H*–H* coupling, which possesses a barrier of 3.50 eV (Figure 2b). Note that no barrier is found for CH_3_CH_2_• dehydrogenation, which is reconfirmed by nudged elastic band (NEB) calculations (Figures S2 and S3), leading to the CH_3_CH_2_• pathway being more favored (Figure 2b) than the pathway of CH_3_CH_2_* with the barrier of 1.25 eV for CH_3_CH_2_* dehydrogenation (Figure 2c). Similarly, the CH_3_CH_2_• pathway is also found to be preferred on SAPO-34 over the CH_3_CH_2_* pathway due to the difficulty of CH_3_CH_2_• adsorption to form CH_3_CH_2_* (Figure 2e). The rate-limiting step of EDH over SAPO-34 is the first dehydrogenation of CH_3_CH_3_(*g*) (1.70 eV) (Figure 2f). Interestingly, a higher rate-limiting barrier is observed over Fe/SAPO-34, indicating a lower catalytic activity for EDH. However, this is not consistent with the experimental result.^21^ Then, how can we understand these conflict observations between experimental results and the theoretical ones? We address this question below.

### Coverage Effects of Adsorbed H* on the Elementary Steps over Fe/SAPO-34 and SAPO-34

The coverage plays a vital role in heterogeneous catalysis, which has been shown to have a significant impact on both adsorption energies and reaction barriers.^26,27,44^ Therefore, the coverage effect was carefully investigated in this work; namely the adsorption energies and reaction barriers with different numbers of adsorbed H* on the acid sites were calculated. There are four active acid sites (active O) around one central atom (Fe and Si over Fe/SAPO-34 and SAPO-34, respectively), and the full coverage was determined with four adsorbed H* on these active sites, as mentioned in the section of Methods and shown in Figure 1b.

Significant coverage effects on the reaction barriers are observed with different numbers of adsorbed H* (Table 2). Our calculations show that the reaction barriers of R1–R4 increase with the increase of adsorbed H* on the acid sites. It can be understood by the weaker binding of both adsorbed H* and CH_3_CH_2_* with the higher coverage of H*(Table S3), where the weaker binding of adsorbates at higher surface coverages has been widely reported.^45^ A decrease of H*–H* coupling barrier is found with more adsorbed H* on the acid sites and the lower barriers for R1 and R4 are observed over SAPO-34 with more adsorbed H*.

**Table 2:** Reaction barriers of R1–R5 with Different Numbers of Adsorbed H* on the Acid Sites

	Fe/SAPO-34					SAPO-34				
no.	H_0_^a^	H_1_	H_2_	H_3_	H_4_	H_0_	H_1_	H_2_	H_3_	H_4_
R1	1.85	2.10	2.75	3.67		1.70	5.02^b^	4.40^b^	2.94^b^	
R2	0.00	0.00	0.65	2.42		0.00	2.71	2.59	2.47	
R3	0.00^b^	0.00^b^	0.97	1.73		0.00^b^	1.21^b^	1.31	1.81	
R4	0.55	1.25	1.36			1.22	0.85	0.00^b^		
R5			3.50	2.03	1.32			0.22	0.00^b^	0.00^b^

a *i* of H_*i*_ refers to the quantity of adsorbed H* existed initially on the acid sites.

b No additional reaction barriers. Energy changes from the initial state to the final state are displayed in Figures S2 and S3.

Because of the energy changes affected by the coverage of H* on the acid sites, the rate-limiting steps and the rate-limiting energies at the steady state with specific H* coverage rather than those at the low coverage traditionally used need to be determined. The calculation of the steady state is of great importance, which can be determined by coverage-dependent microkinetic modeling and will be discussed below.

### Adsorbate–Adsorbate Interactions

Coverage-dependent kinetic calculations were reported in several works.^26,27^ On metal catalysts, the H* coverage effects on adsorption energies may be very small (Figures S4–S6), but they were found to have great influences on the kinetics of the systems. To the best of our knowledge, no work of coverage-dependent microkinetic modeling was reported for catalytic reactions in zeolites (Figure S7). Therefore, a strategy of kinetic calculations for EDH with coverage effects in zeolites is required. In this work, the adsorbate–adsorbate interactions from adsorbed H* were included for both adsorbates (H* and CH_3_CH_2_*) and the transition states (R1, R3, R4, and R5 in Table 1). Note that H* was considered to be the major adsorbate, which was confirmed by the self-consistent result from microkinetic modeling (see activity results with different coverages at 873 K over Fe/SAPO-34 and SAPO-34 in Tables S6–S9).

**Figure 3:**
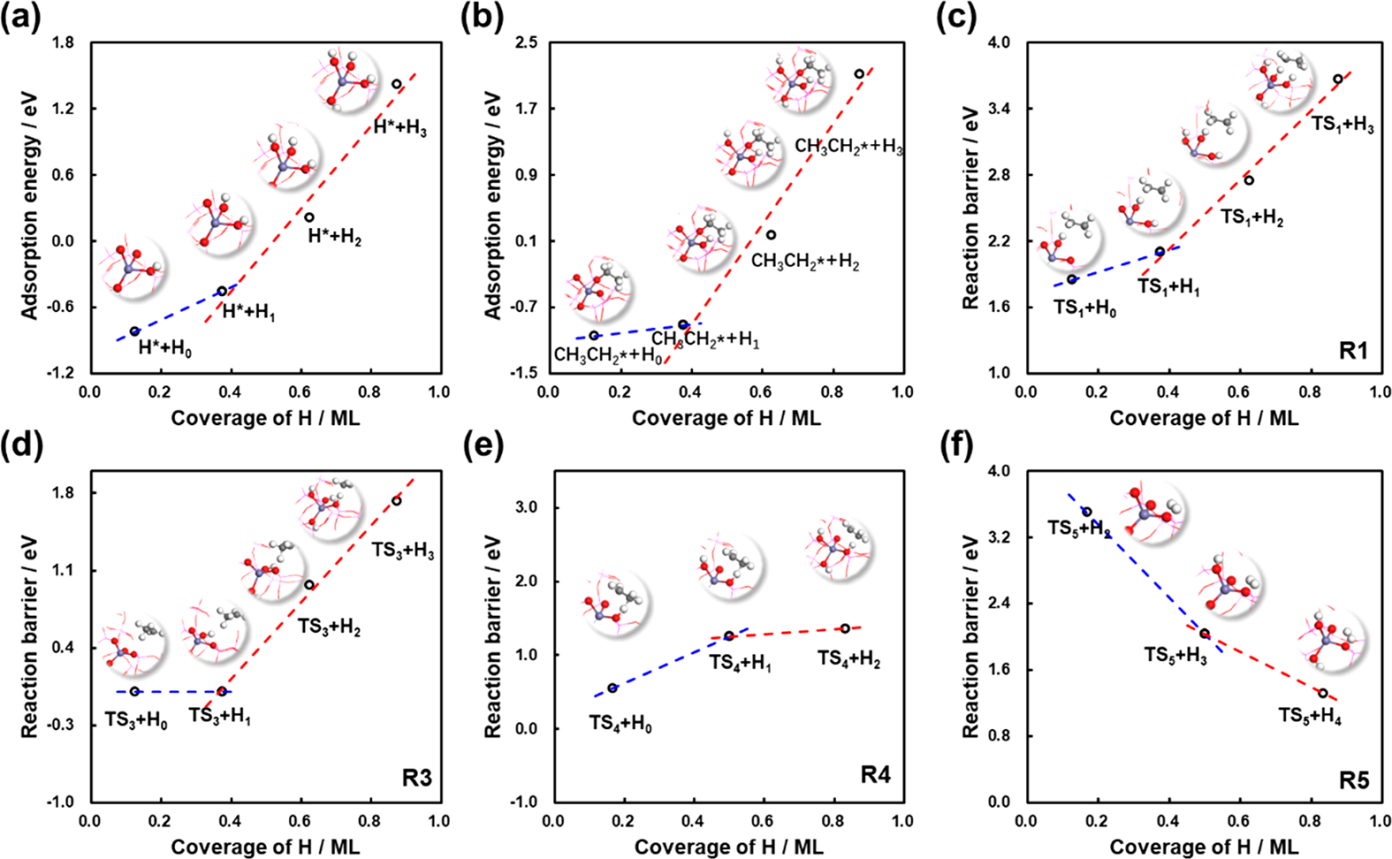
Coverage effects from the adsorbed H* with different surface coverages over Fe/SAPO-34 on the adsorption energies of (a) H* (self-interactions, H*/H_i_), (b) CH_3_CH_2_* (cross-interactions, CH_3_CH_2_*/H_i_), and (c–f) the reaction barriers of R1, R3, R4, and R5. All the adsorption structures and the transition state structures are illustrated in the figure, where *i* in H_*i*_ refers to the number of adsorbed H* existing on the acid sites.

Similar to our previous work,^26,27^ the differential chemisorption energies and the reaction barriers display linear functions of coverages and hence the linear approximations were used to describe the energy changes from the adsorbate–adsorbate interactions over Fe/SAPO-34 (Figure 3) and SAPO-34 (Figure S8), where the coverage effects at low coverages and high coverages are described by two different lines, namely, the two-line model.^26^ Accordingly, the differential adsorption energies of different adsorbates (H* and CH_3_CH_2_*) and the reaction barriers of elementary steps (R1, R3, R4, R5) at different coverages were systematically calculated (see details in method and equation 9). Moreover, the coverage-dependent reaction energies (Δ*G*_1_–Δ*G*_5_) for the elementary steps (R1–R5 in Table 1) were also calculated (see details in method and eqs 10–14.

### Coverage-Dependent and Coverage-Independent Microkinetic Modeling

In this work, the reaction rates were calculated by the microkinetic models based on the kinetic equations of the elementary steps in the nonoxidative dehydrogenation of ethane (Table S5). As mentioned above, the radicals do exist as the key intermediate in EDH. More importantly, after the TS of the radical formation, the radical moves away from the active site. Namely, it should desorb into the gas phase (the free space in the pore in zeolite in this work) once formed. Thus, the readsorption of the radical must be considered in order to continue the reaction. However, the desorption/readsorption of radicals was hardly considered in the traditional kinetic model in heterogeneous catalysis.

**Figure 4:**
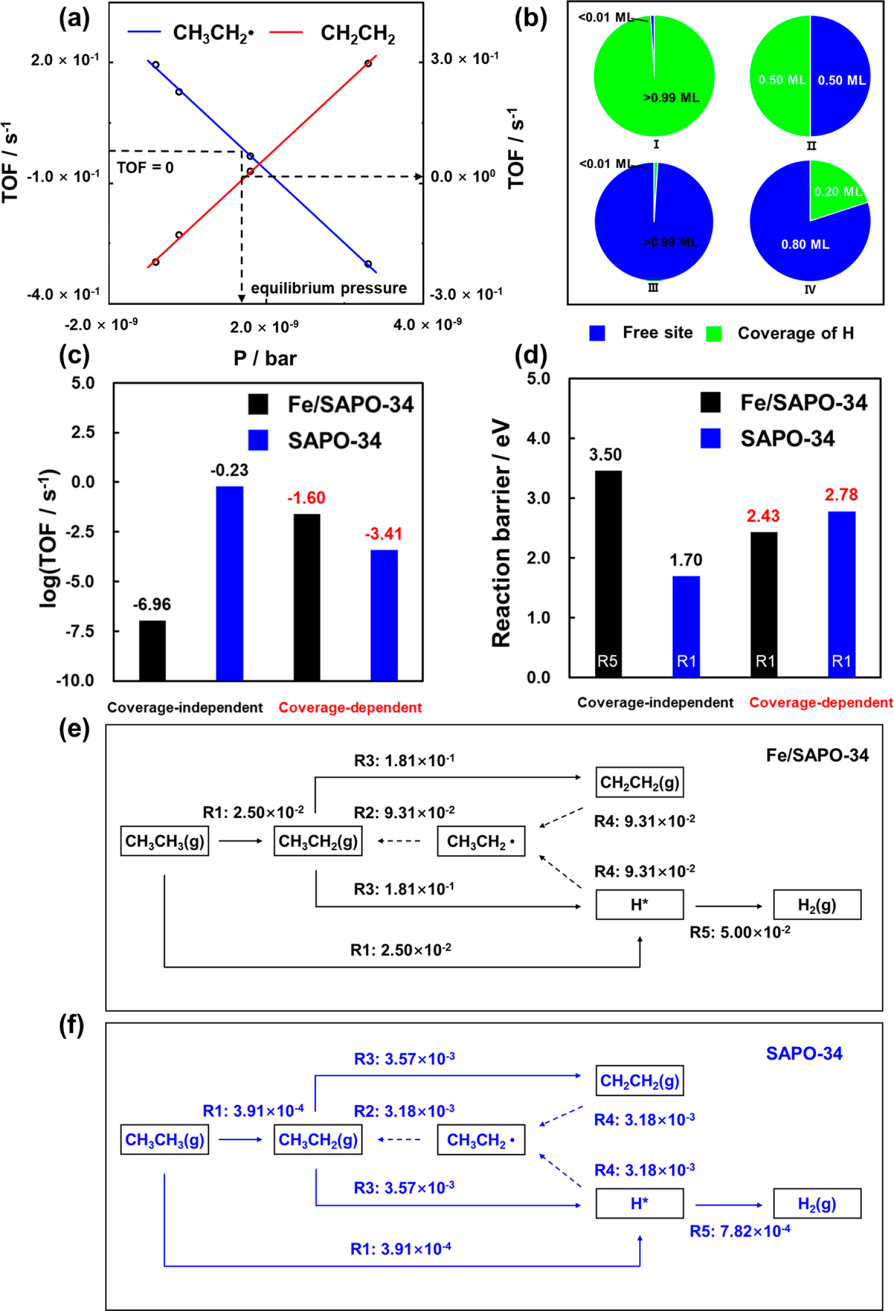
(a) Production rate of ethyl radical as a function of its partial pressures over Fe/SAPO-34 from kinetic simulations. The partial pressure change of CH_3_CH_2_• was used to determine the steady state in the microkinetic simulation. (b) Surface coverage results of (I) coverage-independent microkinetic modeling on Fe/SAPO-34, (II) coverage-dependent microkinetic modeling on Fe/SAPO-34, (III) coverage-independent microkinetic modeling on SAPO-34, and (IV) coverage-dependent microkinetic modeling on SAPO-34. (c) Reaction rate (TOF) from microkinetic modeling. (d) Reaction barriers of the rate-determining steps. (e, f) Flow rate diagrams of EDH over Fe/SAPO-34 and SAPO-34 based on coverage-dependent microkinetic modeling. Microkinetic analyses were performed with the reaction temperature of 873 K, and the pressure of CH_3_CH_3_, CH_2_CH_2_, and H_2_ as 0.3, 0.075, and 0.075 bar, respectively (considering 25% conversion under experimental conditions).

Then, how can one locate the steady state with the inclusion of the desorption/readsorption of radicals in the system? To this end, we considered that an equilibrium between consumption and production of CH_3_CH_2_• radical should be reached at the steady state. Namely, an appropriate partial pressure of CH_3_CH_2_• corresponding to the partial pressure change of CH_3_CH_2_• being zero should be found. To locate the CH_3_CH_2_• pressure at the steady state, kinetic calculations based on a series of the CH_3_CH_2_• pressures were conducted. A linear relation was found between the consumption/production rate and the pressure (Figure 4a). The CH_3_CH_2_• pressure at the equilibrium of consumption and production can be obtained, at which the net rate of CH_3_CH_2_• is equal to 0. As a result, the reaction rate, described by the formation rate of CH_2_CH_2_, can be determined (Figure 4a). It should be noted that the kinetic simulations must reach self-consistencies (Figure S9). Namely, if the output coverage is the same as the input coverage, then the self-convergence is reached.^26,27^ Over Fe/SAPO-34 and SAPO-34, the steady-state pressures of CH_3_CH_2_• were calculated to be 1.32 × 10^–9^ bar and 1.02 × 10^–9^ bar from coverage-dependent kinetic modeling (Figures 4a and S10; Table S11), respectively.

It is worth comparing detailed kinetic results from both coverage-independent modeling and coverage-dependent one for EDH over Fe/SAPO-34 and SAPO-34 (Figure 4). On the one hand, all the active acid sites adsorbed by H* were obtained at the steady states from the coverage-independent kinetic simulations over Fe/SAPO-34 (Figure 4b), which is in conflict with the input coverages in the kinetic calculations. Namely, they were not self-consistent. In addition, the turnover frequencies (TOFs) from Fe/SAPO-34 and SAPO-34 were calculated to be 1.09 × 10^–7^ and 5.92 × 10^–1^ (Figure 4c, Tables S6 and S8), respectively, failing to describe experimental catalytic activity trend (i.e., Fe/SAPO-34 was found to more active than SAPO-34 experimentally). On the other hand, more reasonable results can be found from the coverage-dependent calculations. Half of the active acid sites are observed to be occupied by adsorbed H* over Fe/SAPO-34 at the steady state, while the adsorbed H* over SAPO-34 is obtained to be 0.20 ML. The formation rates of CH_2_CH_2_(*g*) in EDH were calculated to be 2.50 × 10^–2^ and 3.91 × 10^–4^ s^–1^ over Fe/SAPO-34 and SAPO-34 (Figure 4c, Table S7 and Table S9), respectively. These results are in a good agreement with that in the experiment, where a promotion was reported by doping Fe in the zeolite.^21^

Focusing on the reaction mechanism, the first dehydrogenation (R1, 2.43 eV) was found to show the highest barrier for EDH over Fe/SAPO-34 at the steady state (Figure 4d) using the coverage-dependent model, while the H*–H* coupling (R5, 3.50 eV) was determined to be the slowest step using the coverage-independent model. It indicates not only a change of the limiting barrier but a shift of RDS with the rigorous consideration of surface coverage. They were further confirmed to be the rate-determining steps by reversibility and degree of rate control (DRC) (see the reversibility results in Figure S11 and DRC in Figure S12). On the contrary, the first dehydrogenation is always found to be the rate-determining step for EDH over SAPO-34 regardless of the coverage-dependent model or the coverage-independent model used. However, at the steady state the limiting barrier of R1 (2.78 eV) from the coverage-dependent modeling is higher than that (1.70 eV) from the coverage-independent modeling. In addition, the CH_3_CH_2_• pathway makes the largest contribution to the total rate (Figure 4e) over Fe/SAPO-34, which is also favored over SAPO-34 (Figure 4f), suggesting quantitatively the result that the CH_3_CH_2_• pathway is the major pathway in the system.

### Quantitative Comparison

**Figure 5:**
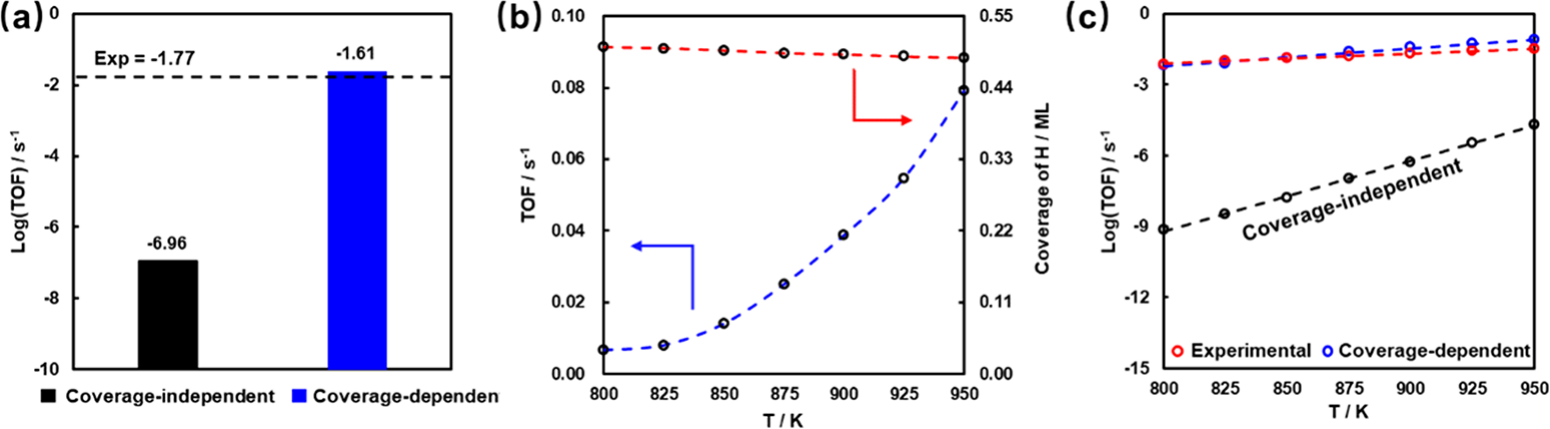
(a) Comparison of different kinetic modellings with experimental values at 873 K. (b) Theoretical rates (TOF, blue dash line) and H* coverage at different temperatures from the coverage-dependent kinetic modeling. (c) Comparison between estimated experimental reaction rates (red) and theoretical reaction rates (blue) with the temperatures from 800 to 950 K.

As discussed above, more reasonable results are obtained from the coverage-dependent kinetic simulations. Although no experimental work was carried out on the same zeolites, we found an experimental work on a similar zeolite to one in our work, in which the adsorption energy of H (−0.80 eV) is almost identical to our one (−0.81 eV). To make a direct comparison possible, we have estimated the TOF values (see more details in Note 2 of SI). Figure 5a shows the experimental TOF (1.68 × 10^–2^) is close to the theoretical TOF based on the coverage-dependent kinetic modeling (2.50 × 10^–2^) at 873 K, while it is far from coverage-independent ones (1.09 × 10^–7^).

More importantly, kinetic calculations were carried out with temperatures from 800 to 950 K. Figure 5b shows the trend of theoretical rates with the increase of temperature and the corresponding change of acid site coverages. It is interesting to note that the barrier of the rate-determining step, namely the first dehydrogenation, increases with temperature (see the activity results with the corrections of adsorbate–adsorbate interactions under 800–950 K for Fe/SAPO-34 in Table S10). However, the total rate still increases as the temperature is raised, while the acid site coverage remains quite constant, indicating that the acid site coverage is less sensitive to the temperature at this stage (see more details in Note 3 of the SI).

In addition, to further verify the accuracy of the result from the coverage-dependent scheme, the comparison between estimated experimental reaction rates (red, see Note 2 of the SI) and theoretical reaction rates (blue) with the temperatures from 800 to 950 K was performed (Figure 5c), and an excellent agreement between theoretical and experimental trends can be clearly seen from the figure: The largest differences are smaller than 0.06 s^–1^ at 950 K. It displays a giant improvement of almost 7 orders of magnitude from the result of the coverage-independent ones. These results demonstrate that the coverage effect is not only vital in the investigation of metal surface kinetics, but also crucial for the reactions in zeolites. In other words, the microkinetic calculations in zeolites also needs to include the coverage effect to achieve the accurate results. It is worth noting that there is only one Fe atom in the center of active acid sites, but all the first neighbor O sites (acid sites) must be considered in order to obtain the correct kinetic results.

### Discussion on the Drawback of Traditional Model in Heterogeneous Catalysis

As mentioned in the Introduction, in the traditional model, any catalysts are described as open surfaces, which means that any intermediates desorbed from the surfaces will have an extremely low probability to readsorb on the surface. This is because the coverage of any intermediate is typically very low; then due to the nature of open surfaces, thus resulting in a large volume above the surface, they should possess extremely low partial pressures if they desorb. Consequently, the partial pressures of the desorbed intermediates will be far too low for them to have a reasonable chance to readsorb on the surface. If one is to carry out a kinetic calculation, the rates of readsorption of the intermediates should be so small that they could not significantly affect the kinetics of the system. Thus, the readsorption of intermediates could be omitted in the kinetic simulation if an open surface model, i.e. the traditional model, is used.

**Figure 6:**
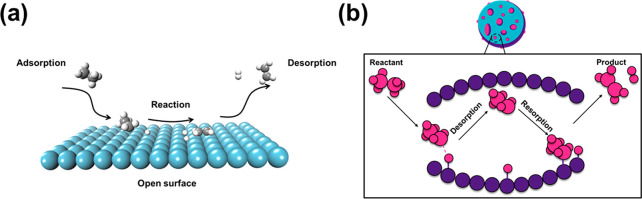
(a) Traditional model, i.e., the open surface model without any confinement effect, to describe catalytic cycles in heterogeneous catalysis, in which the surface reactions exclusively occur on the surface. (b) More realistic model possessing reactive sites and also local porous structures.

Quantitatively, the traditional model (Figure 6a) cannot be used to obtain the correct kinetics for our system: After the transition state, the formed CH_3_CH_2_• radical desorbs immediately to the gas phase and then the steady state could not be achieved because of too low partial pressure of CH_3_CH_2_• radical if the open surface model was applied, which would be detrimental to the kinetics. In order to verify the method, we have added more comparison between our theoretical work and experimental work as follows (Figure S14).

(i) Considering the very large volume above the surface because of the nature of the open surface, a very low CH_3_CH_2_• pressure (10^–50^ bar) is applied to approximate an open surface model, and then a consumption rate of 2.60 × 10^–1^ s^–1^ of CH_2_CH_2_• was obtained. This is in fact the reverse reaction of the real reaction interested, indicating a fatal weakness of the open surface model in describing the reaction system.

(ii) By contrast, if the kinetics is solved self-consistently using our approach in which finite size pores exist, the formation rate of ethene is 2.50 × 10^–2^ s^–1^ at 873 K, which is very close the experimental value (1.68 × 10^–2^ s^–1^).

It confirms the importance of the existence of finite size pores in heterogeneous catalysis discussed above. One may question whether our specific system is general by arguing that the zeolites are special catalysts. It should be noted that both metals and oxides are successfully used as catalysts for the ethane dehydrogenation, in which the CH_3_CH_2_• radical is an important intermediate.^23,46^ From the transition state of CH_3_CH_2_• radical formation, it is expected that the CH_3_CH_2_• radical would desorb into the gas phase on metal and oxide catalysts.^24,47^ How can one rationalize this catalytic result? We suggest that both metal and oxide catalysts may contain pore structures, resulting in the CH_3_CH_2_• radical desorbing into the pores to maintain certain partial pressures in the systems, which are similar to the zeolites we studied here. In fact, most catalysts are prepared with pore structures on purpose. We argue that even in extreme cases where “no” pore exists in catalysts, it remains challenging on evaluating partial pressure of radicals in those systems.

Therefore, the pore structures of catalysts may be much more common, and we argue that it is a key structural characteristic of any catalysts regardless of metals, oxides and zeolites. We suggest that a more general model for catalytic reaction in heterogeneous catalysis (Figure 6b), in which there are many pore structures in the catalyst, which provide local confined environments^48,49^ for catalytic reactions. The central concept emphasized here may have a profound impact on heterogeneous catalysis; some puzzling results in the literature may be readily explained using the concept discussed in this work. For example, some interesting results were reported to relate the morphologies of catalysis,^50^ which could be linked to some special pore structures.

It is noted that the existence of pore structures in catalysts is well-known. In particular, the pore structures in zeolites were extensively discussed before; hence, our discussion might seem to be nothing new. However, the pore structures in zeolites were mainly discussed with the context of shape selectivity for products or reactants in the literature. It is worth stressing that in our work it is discussed with the context of feasibility of kinetic description of the systems. Our work provides the first quantitative evidence that the kinetics of the catalytic system is not feasible without pore structures, to the best of our knowledge. Without the kinetic feasibility, any catalytic system is meaningless. Considering that real catalysts might always contain local pore structures, this concept may well be general and may be of paramount importance in heterogeneous catalysis.

## Conclusion

This work represents the first attempt to perform a full kinetic simulation with the coverage effect over zeolites. The dehydrogenation of ethane to ethylene (EDH) was selected as the model reaction in a zeolite incorporated by a metal atom (Fe/SAPO-34) and the same set of calculations were also carried out in pristine SAPO-34 zeolite for comparison. First, the reaction pathways and reaction barriers were calculated in detail at low coverage and a coverage-independent kinetic simulation was carried out. Second, a coverage-dependent self-consistent kinetic modeling was established based on the calculated DFT energies, in which adsorbate–adsorbate interactions are considered, including adsorption energies and reaction barriers. Based on the above calculations, the following conclusions are reached:

(i) The rate-limiting step of EDH over Fe/SAPO-34 from the coverage-independent modeling is the H*–H* coupling, possessing a barrier of 3.50 eV, while it has changed to the first dehydrogenation with a barrier of 2.43 eV based on the coverage-dependent modeling. The rate-limiting step over SAPO-34 is the first dehydrogenation with the barrier of 1.70 eV from the coverage-independent model. While the rate-limiting step is still the first dehydrogenation, the barrier is increased to 2.78 eV based on the coverage-dependent modeling.
(ii) Two distinguish reaction channels were identified at the low coverage: the CH_3_CH_2_• pathway (R1 + R3 + R5) and the CH_3_CH_2_* pathway (R1 + R2 + R4 + R5) over both Fe/SAPO-34 and SAPO-34 (R1–R5 are defined in the main text). Over Fe/SAPO-34, the CH_3_CH_2_• pathway was more favored than the CH_3_CH_2_* pathway, which is also the case for SAPO-34.
(iii) The active acid sites are almost completely covered by H over Fe/SAPO-34 and SAPO-34 using the coverage-independent kinetic modeling at the steady states, and the catalytic rates are 1.09 × 10^–7^ and 5.92 × 10^–1^ s^–1^, respectively. However, only half of the acid sites were observed to be occupied by adsorbed H* over Fe/SAPO-34 at the steady state using coverage-dependent kinetic modeling, while the adsorbed H* coverage of 0.20 ML was obtained over SAPO-34.
(iv) Using the coverage-dependent kinetic modeling, the total rates were calculated to be 2.50 × 10^–2^ and 3.91 × 10^–4^ s^–1^ for Fe/SAPO-34 and SAPO-34, respectively, which are significantly higher than those from the coverage-independent one, showing that Fe/SAPO-34 possesses much higher catalytic activity than that of SAPO-34.
(v) The estimated experimental TOF (1.68 × 10^–2^) is close to the theoretical one obtained from the coverage-dependent kinetic modeling (2.50 × 10^–2^) at 873 K, while it is far from coverage-independent ones (1.09 × 10^–7^). If one is to obtain accurate results to link theoretical data with experiment, the effect of coverage must be considered in the kinetics in zeolite systems.

Finally, the following insights into fundamental models in heterogeneous catalysis are obtained:

(i) The traditional model (i.e., the open surface model) to depict catalytic reactions cannot be applied to the kinetics of our system. The open surface model is lack of the confinement effect, leading to the probability of intermediates readsorption being extremely low.
(ii) A more general model should contain reaction sites (i.e., the adsorption/reaction sites) and also local pore structures that are vital to describing the kinetics of many real catalytic reactions.

## Supporting Information Available

Activation barriers and reaction free energies; NEB diagram of energy change; microkinetic kinetic details; and thermodynamic corrections (PDF)
