# Supplementary material for: Quantitative Evidence to Challenge the Traditional Model in Heterogeneous Catalysis: Kinetic Modeling for Ethane Dehydrogenation over Fe/SAPO-34
Source: JACS Au. 2022 Dec 19;3(1):165–75. doi: 10.1021/jacsau.2c00576 (PMC9875371; doi:10.1021/jacsau.2c00576)
Supplement: Supplementary file 1 — au2c00576_si_001.pdf [file au2c00576_si_001.pdf]

Supporting information for

Quantitative Evidence to Challenge the Traditional Model in Heterogeneous Catalysis: Kinetic Modelling for Ethane Dehydrogenation over Fe/SAPO-34

Peng Chen[1], Ying Liu[1], Yarong Xu[2], Chenxi Guo[3,*], and P. Hu[1,4,*]

[1] Key Laboratory for Advanced Materials, Centre for Computational Chemistry and Research Institute of Industrial Catalysis, East China University of Science and Technology, 130 Meilong Road, Shanghai 200237, China

[2] Research Institute of Urumqi Petrochina Chemical Company, Urumqi 83000, China

[3] Department of 5T Technology, Zhejiang SUPCON Technology Co., Ltd., Hangzhou 310053, China

[4] School of Chemistry and Chemical Engineering, The Queen's University of Belfast, Belfast BT9 5AG, United Kingdom

* Email: guochenxi@supcon.com
* Email: p.hu@qub.ac.uk

Table of Contents

Supporting Figures S2-S15

Supporting Tables S16-S28

Supporting Notes S29-S31

Supporting References S32

Supporting Figures

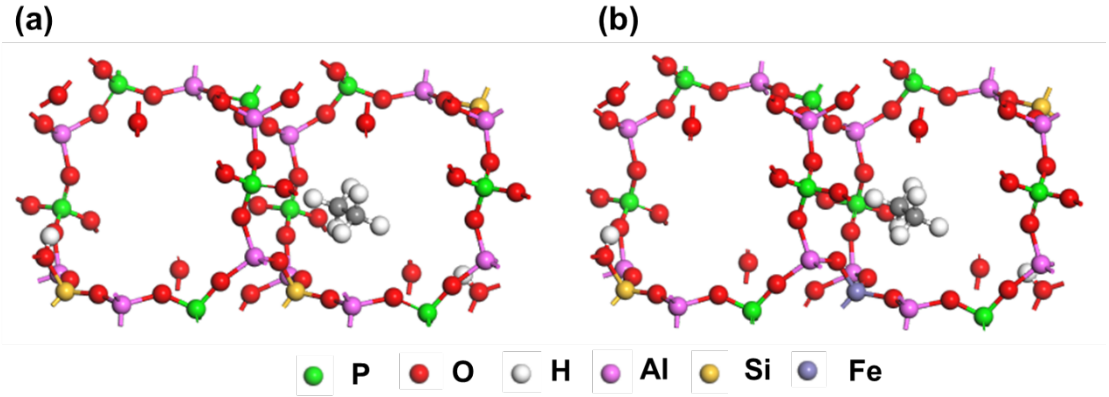

Figure S1. Structural illustrations of ethane adsorption in the channel of (a) SAPO-34 and (b) Fe/SAPO-34.

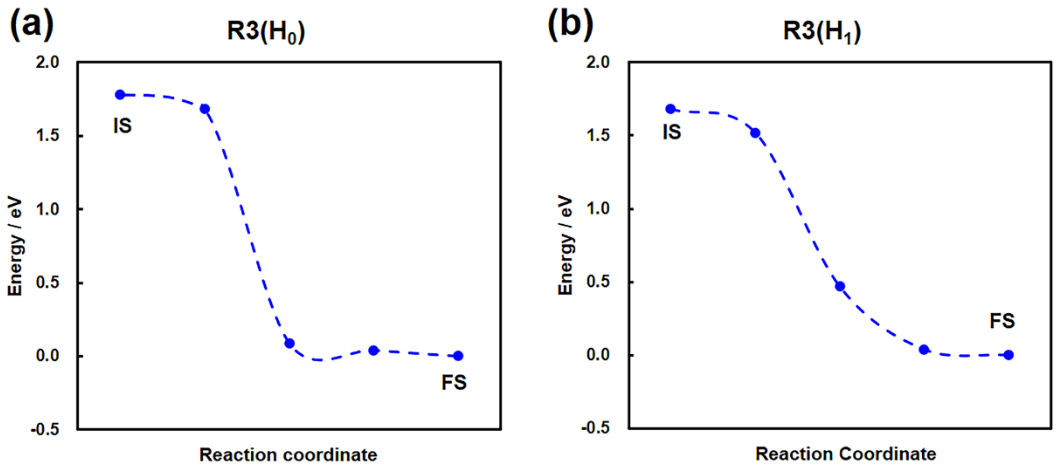

Figure S2. Diagrams of energy changes from the initial states to the final states over Fe/SAPO-34: (a) for R3 with H_0 and (b) R3 with H_1, where H_0 and H_1 refer to the numbers of 1 and 2 adsorbed H* on the acid sites, respectively.

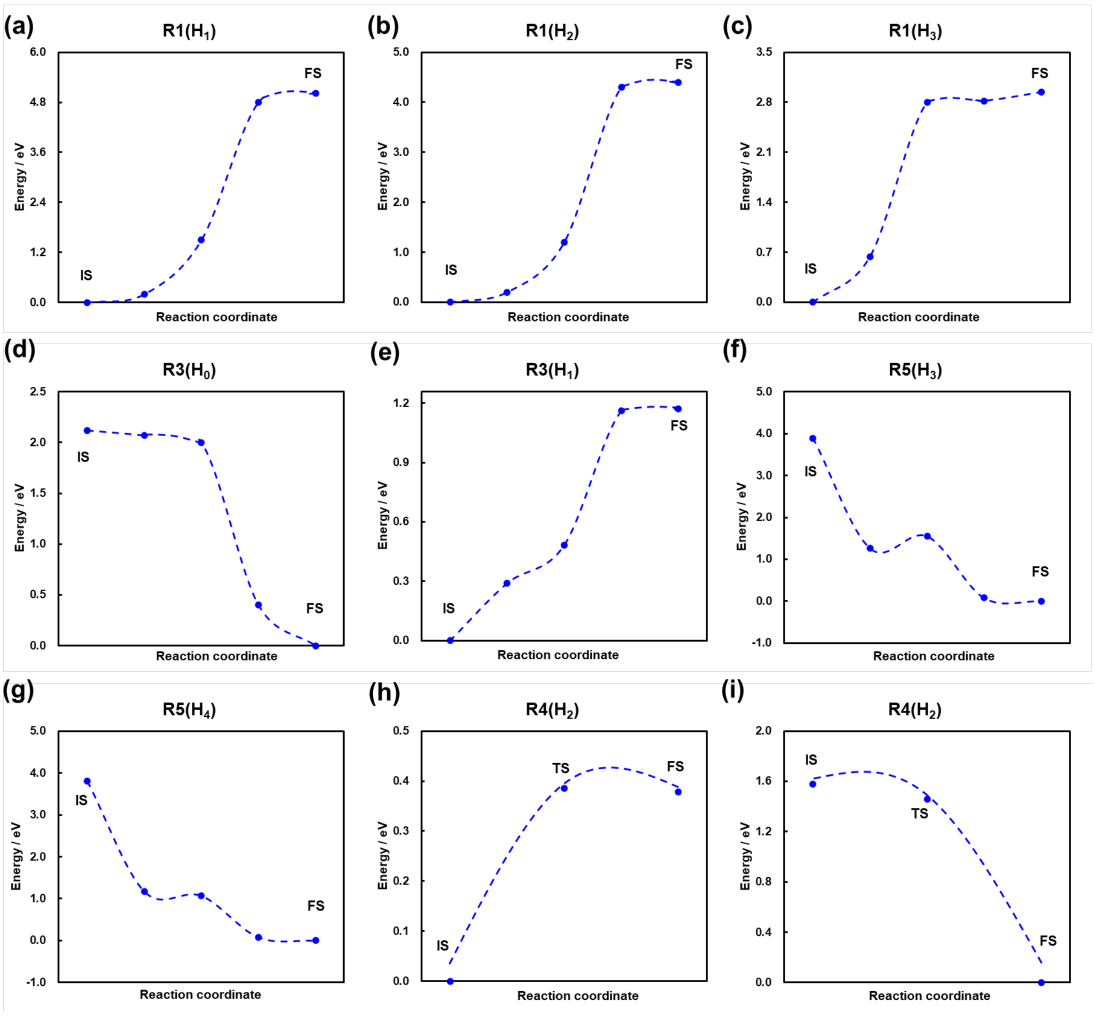

Figure S3. Diagrams of energy changes from the initial states to the final states over SAPO-34 for elementary steps: (a), (b) and (c) for R1; (d) and (e) for R3; (f) and (g) for R5; (h) and (i) for R4, where i of H_i refers to the number of adsorbed H* on the acid sites. The figure in (h) shows the energy changes of R4 at 0 K. The figure in (i) shows the energy changes of R4 at 873 K with the free energy correction (see the main text). The transition states (TS) of R4 were determined by the constrained optimization, resulting in an energy barrier at 0 K. However, no free energy barrier was found after the free energy correction at 873 K.

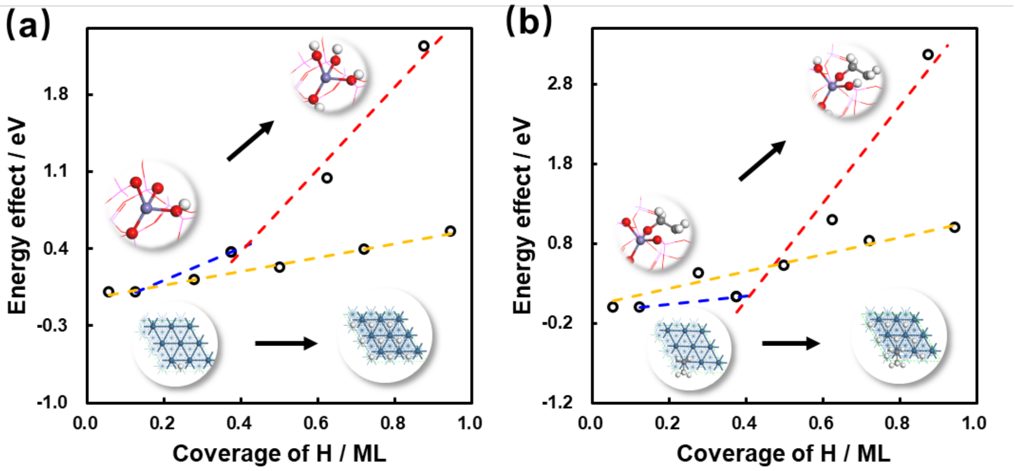

Figure S4. Coverage effects of adsorbed H* over Fe/SAPO-34 and Pt(111) on the adsorption energies of (a) H* (self-interactions, H*/H_{sur}), (b) CH_3CH_2* (cross-interactions, CH_3CH_2*/H_{sur}), where the coverage of H_{sur} is varied. Adsorption structures are illustrated in the figure.

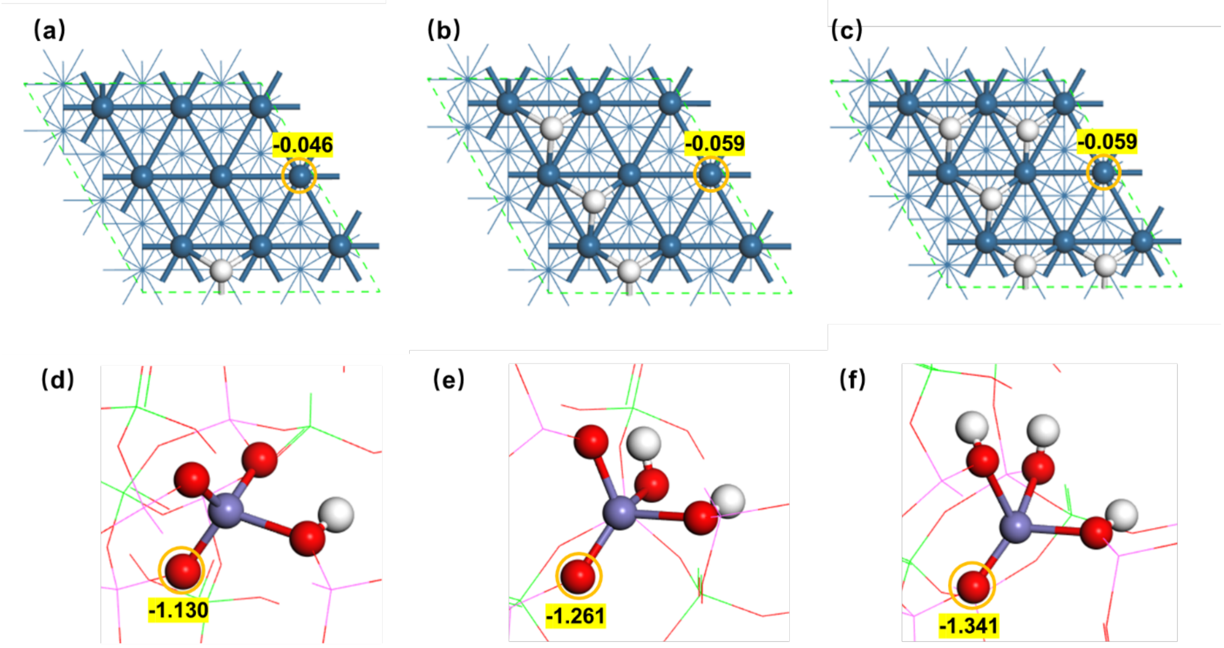

Figure S5. Bader charge analyses on the H_{sur} coverage effects on H* (self-interactions, H*/H_{sur}) (a-c) on Pt(111) and (d-f) on Fe/SAPO-34. In each figure, the orange circle shows a Pt atom near H on Pt(111) and an oxygen atom near H on Fe/SAPO-34, respectively, and the charge is the black numbers with yellow background.

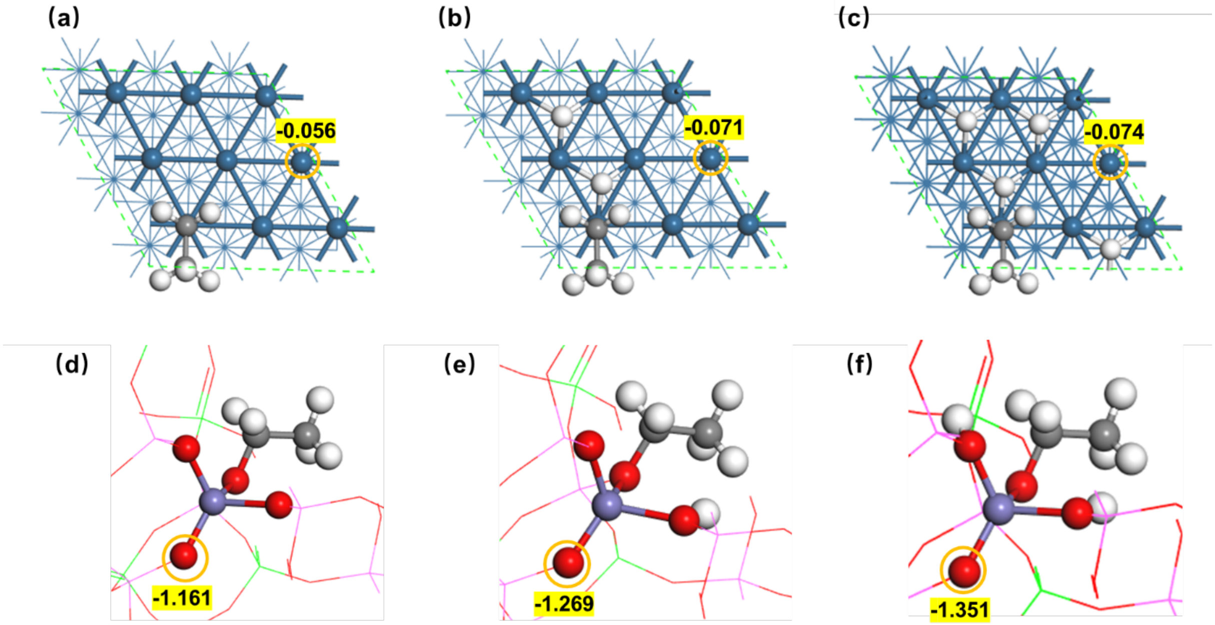

Figure S6. Bader charge analyses on H_{sur} coverage effects on CH_3CH_2* (cross-interactions, CH_3CH_2*/H_{sur}) (a-c) on Pt(111) and (d-f) on Fe/SAPO-34. In each figure, the orange circle shows a Pt atom near H on Pt(111) and an oxygen atom near H on Fe/SAPO-34, respectively, and the charge is the black number with yellow background.

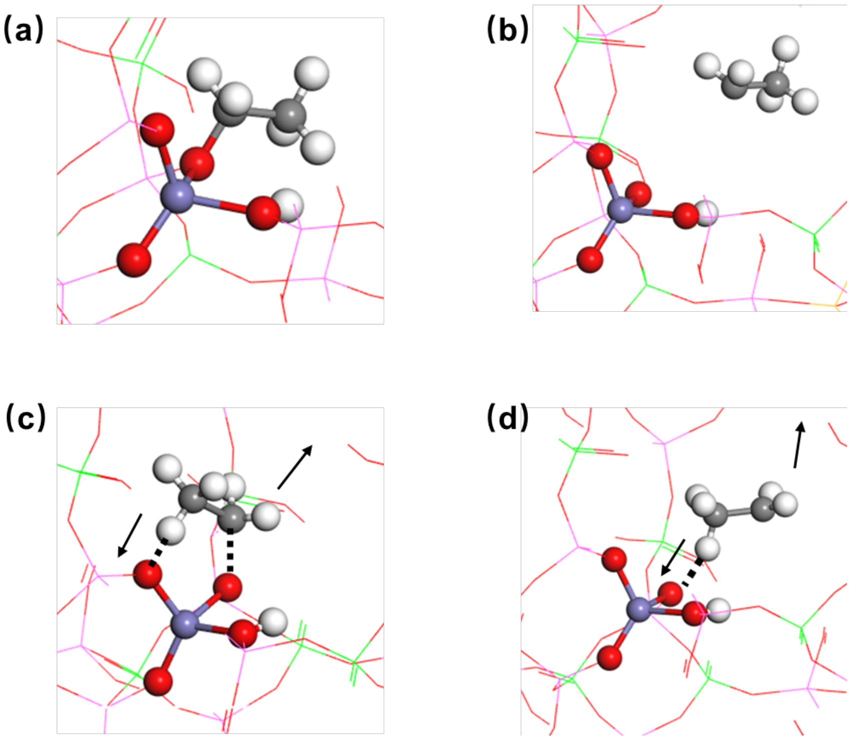

Figure S7. Initial state structures of CH_3CH_2* (a) and CH_3CH_2• (b) in the zeolite system. The transition state structures of their dehydrogenation reactions are illustrated in (c) and (d), respectively.

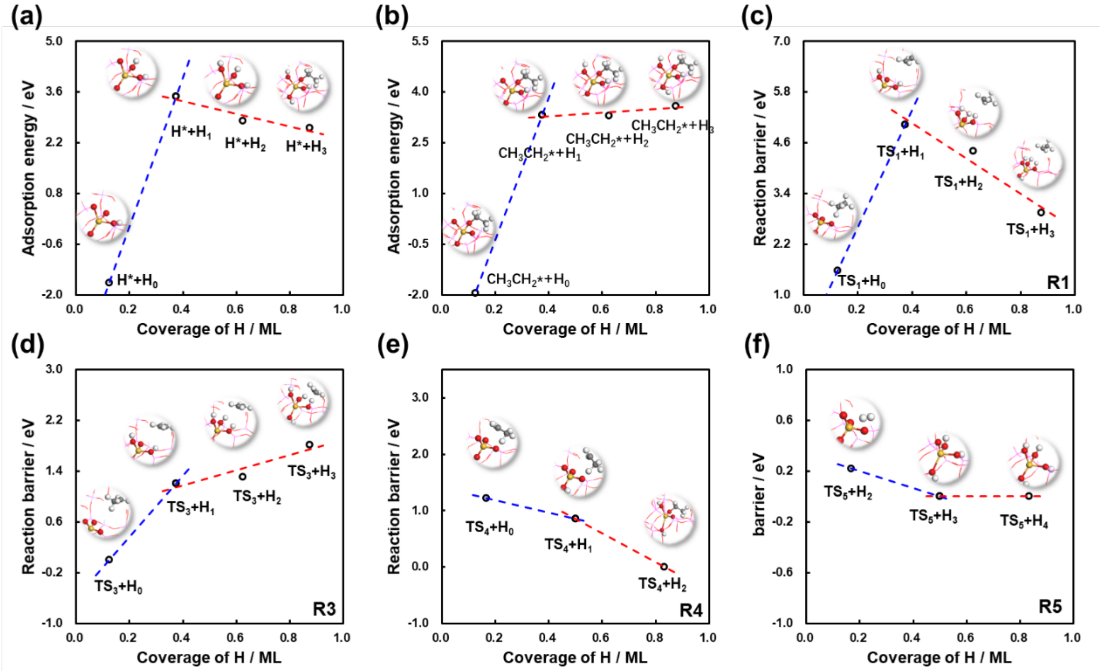

Figure S8. Coverage effects from the adsorbed H* with different surface coverages over SAPO-34 on the adsorption energies of (a) H* (self-interactions, H*/H_i), (b) CH_3CH_2* (cross-interactions, CH_3CH_2*/H_i), and the reaction barriers of (c) R1, (d) R3, (e) R4 and (f) R5. All the adsorption structures and the transition state structures are illustrated in the figure, where i in H_i refers to the number of adsorbed H* on the acid sites.

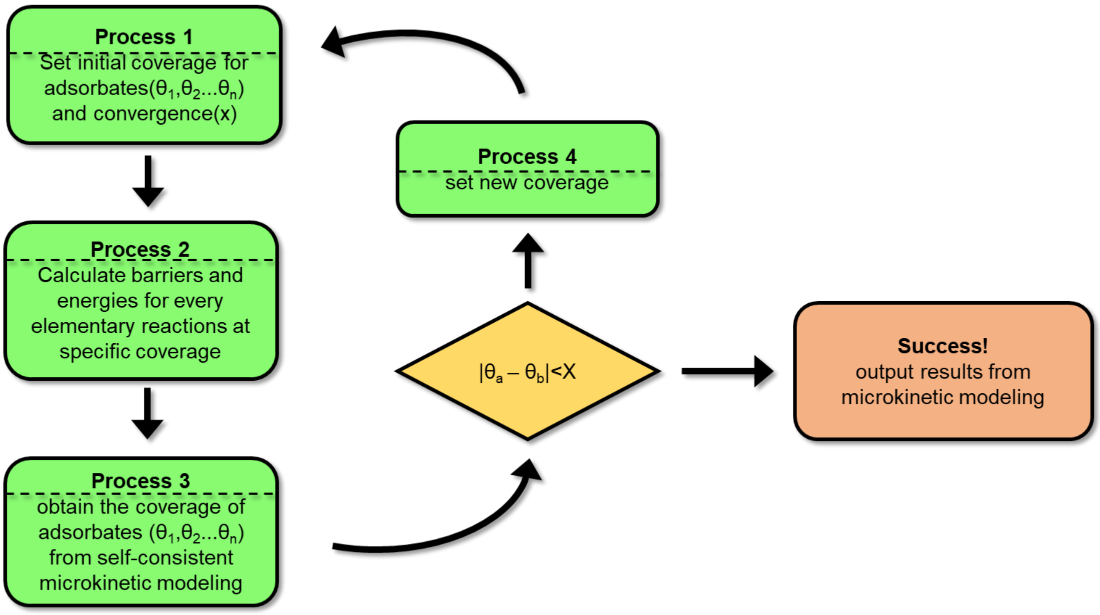

Figure S9. Flow chart for the scheme of the coverage-dependent microkinetic modelling, where θ_a and θ_b represent the initial setting coverage and the coverage calculated from self-consistent microkinetic modelling. X represents the convergence, namely the difference between the initial setting coverage and the calculated one (X = 0.01 in this work).[1] Microkinetic modelling was conducted in this work through CATKINAS.[2,3]

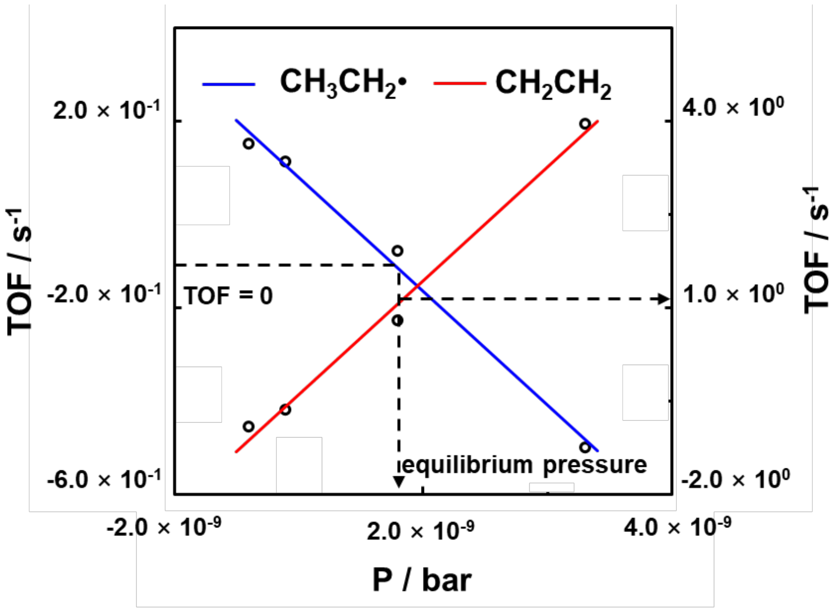

Figure S10. Formation rates of ethyl radical and CH_2CH_2 as a function of ethyl radical pressure over SAPO-34. To determine the CH_3CH_2• pressure at the steady state, kinetic calculations based on a series of the CH_3CH_2• pressures were conducted. A linear relation was found between the consumption/production rate and the pressure. The CH_3CH_2• pressure at the equilibrium of consumption and production can be obtained, at which the net rate of CH_3CH_2• is equal to 0.

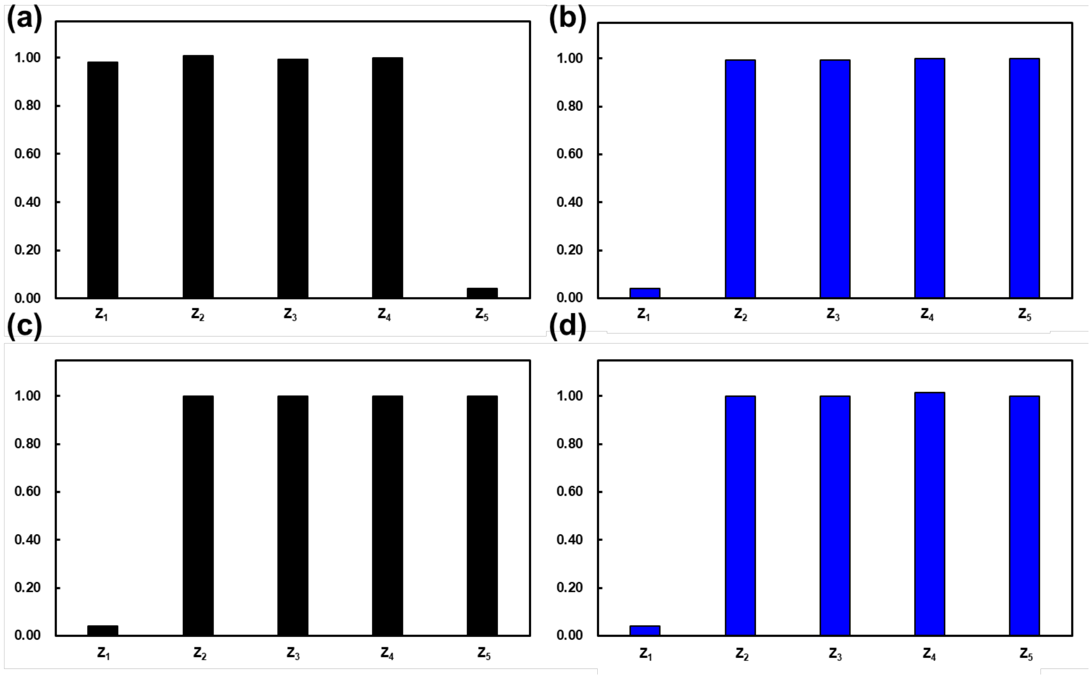

Figure S11. Reversibility (Z_i) of elementary reactions from (a) and (b) the coverage-independent modelling and (c) and (d) the coverage-dependent modelling for Fe/SAPO-34 and SAPO-34, respectively. The black columns represent results from Fe/SAPO-34, and the blue columns represent the results from SAPO-34 and i in Z_i represents elementary step i. Here, the reversibility of an elementary step, Z_i, is defined as the ratio of the reverse rate to the forward rate. The numerical value of the reversibility is equal to 0 for an irreversible step and approaches 1 for a quasi-equilibrated step.

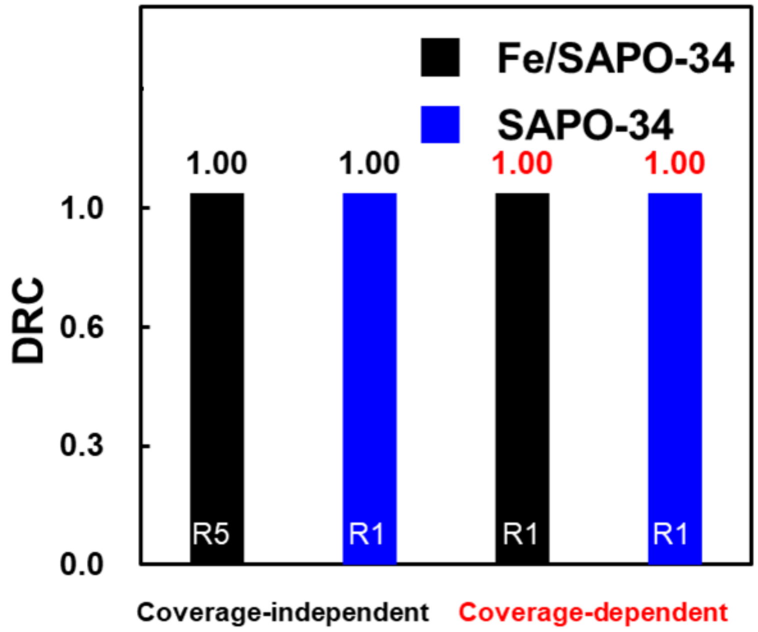

Figure S12. Degree of rate control (DRC) over Fe/SAPO-34 and SAPO-34 from coverage-independent modelling and coverage-dependent modelling. The black columns represent the results from Fe/SAPO-34, and the blue column from SAPO-34. Here, the magnitude of the degree of rate control of each elementary reaction reflects the relative degree of the elementary reaction in controlling the rate of the overall reaction.

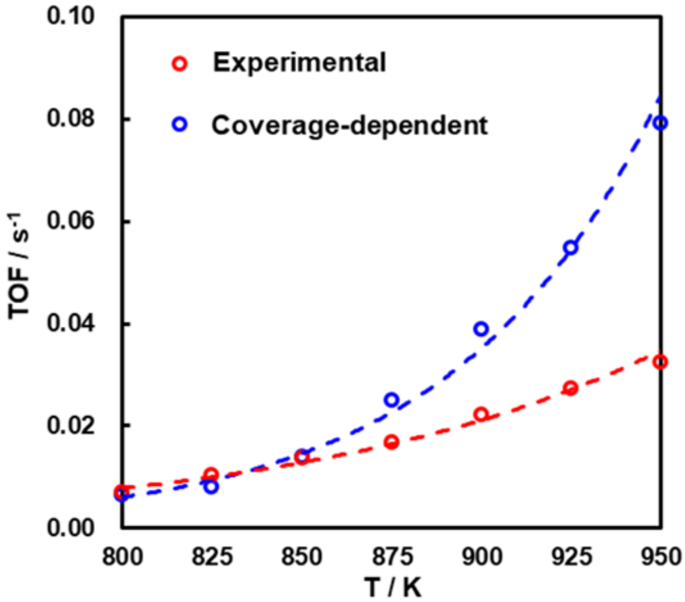

Figure S13. Comparison between estimated experimental reaction rates (red, see Note 2 below) and theoretical reaction rates (blue) with the temperatures from 800 to 950 K. It is noted that the theoretical simulations overestimate the activity at high temperatures. However, the scale of TOF is very small and hence the differences are small.

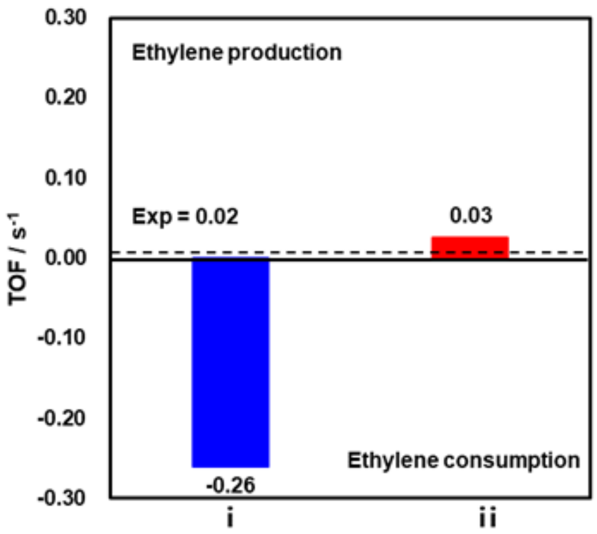

Figure S14. Theoretical rates calculated by microkinetic simulations under different conditions (i refers to a very low pressure of ethyl radical to model an open surface, and ii shows the result at the steady state obtained self-consistently from the coverage-dependent microkinetic simulation). The estimated experimental TOF is also indicated in the figure. Note that the result from our self-consistent kinetic simulations is very close to the experimental one, while the ethylene is consumed under condition i which is the reverse reaction of the real catalytic reaction.

Supporting Tables

Table S1. Adsorption energies (eV) of ethane over Fe/SAPO-34 and SAPO-34.

	**Fe/SAPO-34**	**SAPO-34**
**CH_3CH_3**	-0.25	-0.29

Table S2. Elementary steps for EDH over Fe/SAPO-34 and SAPO-34 with free energy barriers (G_a) and reaction free energies (ΔG) at low coverage.

		**Fe/SAPO-34**		**SAPO-34**	
No.	Elementary steps	G_a (eV)	ΔG (eV)	G_a (eV)	ΔG (eV)
**R1**	$CH_3CH_3(g) + * \rightarrow CH_3CH_2\bullet + H*$	1.85	0.27	1.70	-0.41
**R2**	$CH_3CH_2\bullet + * \rightarrow CH_3CH_2*$	0.00	-0.27	2.71#	2.71
**R3**	$CH_3CH_2\bullet + * \rightarrow CH_2CH_2(g) + H*$	0.00#	-1.60	1.21#	1.21
**R4**	$CH_3CH_2* \rightarrow CH_2CH_2(g) + H*$	1.25	-1.32	0.85	-1.51
**R5**	$2H* \rightarrow H_2(g) + 2*$	3.50	1.37	0.22	-0.75

No additional barriers were found in these elementary steps. Diagrams of energy changes from the initial states to the final states for elementary steps by NEB are displayed in Figure S2-S3.

Table S3. Differential adsorption energies of H* and CH_3CH_2* at different H* coverages over Fe/SAPO-34 and SAPO-34.

	**Fe/SAPO-34**				**SAPO-34**			
Species	H_1[#]	H_2	H_3	H_4	H_1	H_2	H_3	H_4
**H**	-0.81	-0.45	0.21	1.41	-1.66	3.47	2.81	2.60
**CH_3CH_2**	-0.97	-0.92	0.14	2.16	-1.94	3.22	3.29	3.58

[#] Here i in H_i refers to the number of adsorbed H* on the acid sites.

Table S4. Parameters of the two-line model for Fe/SAPO-34 and SAPO-34.

Adsorbate-adsorbate interactions	low coverage region		high coverage region	
	slope(a)	Intercept(b)	slope(a)	Intercept(b)
	Fe/SAPO-34			
H^*/H_{sur}	1.46	-1.00	3.74	-1.94
$CH_3CH_2^*/H_{sur}$	0.50	-1.10	6.07	-3.34
R_1TS/H_{sur}	1.00	1.72	3.14	0.87
R_3TS/H_{sur}	0.00	0.00	3.46	-1.26
R_4TS/H_{sur}	2.10	0.19	0.33	1.08
R_5TS/H_{sur}	-4.41	4.23	-2.13	3.19
	SAPO-34			
H^*/H_{sur}	20.57	-4.24	-1.74	4.05
$CH_3CH_2^*/H_{sur}$	21.05	-4.57	0.52	3.06
R_1TS/H_{sur}	13.76	-0.14	-4.16	6.72
R_3TS/H_{sur}	4.84	-0.60	1.20	0.69
R_4TS/H_{sur}	-1.11	1.40	-2.55	2.12
R_5TS/H_{sur}	-0.66	0.33	0.00	0.00

Table S5. Elementary steps and the corresponding rate equations in our kinetic modelling for non-oxidative ethane dehydrogenation over Fe/SAPO-34, where * represents an active site, and CH_3CH_2• is a CH_3CH_2 radical.

No.	Elementary steps	Rate equations
**R1**	CH_3CH_3(g) + * → CH_3CH_2• + H*	$r_1 = k_1 P_{CH_3CH_3}\theta_* - k_{-1} P_{CH_3CH_2}\theta_H$
**R2**	CH_3CH_2• + * → CH_3CH_2*	$r_2 = k_2 P_{CH_3CH_2}\theta_* - k_{-2}\theta_{CH_3CH_2}$
**R3**	CH_3CH_2• + * → CH_2CH_2(g) + H*	$r_3 = k_3 P_{CH_3CH_2}\theta_* - k_{-3} P_{CH_2CH_2}\theta_H$
**R4**	CH_3CH_2* → CH_2CH_2(g) + H*	$r_4 = k_4\theta_{CH_3CH_2} - k_{-4} P_{CH_2CH_2}\theta_H$
**R5**	2H* → H_2(g) + 2*	$r_5 = k_5{\theta_H}^2 - k_{-5} P_{H_2}{\theta_*}^2$

Table S6. Theoretical rate and surface coverage results calculated from the coverage-independent kinetic modelling at 873 K over Fe/SAPO-34.

No.	Elementary steps	Rate (s^{-1})	Adsorbate	Coverage (ML)
**R1**	$CH_3CH_3(g) + * \rightarrow CH_3CH_2\bullet + H*$	1.09×10^{-7}	H*	9.99×10^{-1}
**R2**	$CH_3CH_2\bullet + * \rightarrow CH_3CH_2*$	2.40×10^{-13}	CH_3CH_2*	1.57×10^{-9}
**R3**	$CH_3CH_2\bullet + * \rightarrow CH_2CH_2(g) + H*$	1.09×10^{-7}	*	7.24×10^{-5}
**R4**	$CH_3CH_2* \rightarrow CH_2CH_2(g) + H*$	2.40×10^{-13}		
**R5**	$2H* \rightarrow H_2(g) + 2*$	1.09×10^{-7}		

Table S7. Theoretical rates and surface coverage results calculated from the coverage-dependent kinetic modelling at 873 K over Fe/SAPO-34.

No.	Elementary steps	Rate (s^{-1})	Adsorbate	Coverage (ML)
**R1**	CH_3CH_3(g) + * → CH_3CH_2• + H*	2.50 × 10^{-2}	H*	4.92 × 10^{-1}
**R2**	CH_3CH_2• + * → CH_3CH_2*	-9.31 × 10^{-2}	CH_3CH_2*	4.01 × 10^{-7}
**R3**	CH_3CH_2• + * → CH_2CH_2(g) + H*	1.18 × 10^{-1}	*	5.07 × 10^{-1}
**R4**	CH_3CH_2* → CH_2CH_2(g) + H*	-9.31 × 10^{-2}		
**R5**	2H* → H_2(g) + 2*	2.50 × 10^{-2}		

Table S8. Theoretical rates and surface coverage results calculated from the coverage-independent kinetic modelling at 873 K over SAPO-34.

No.	Elementary steps	Rate (s^{-1})	Adsorbate	Coverage (ML)
**R1**	$CH_3CH_3(g) + * \rightarrow CH_3CH_2\bullet + H*$	5.92×10^{-1}	H*	1.87×10^{-3}
**R2**	$CH_3CH_2\bullet + * \rightarrow CH_3CH_2*$	2.14×10^{-11}	CH_3CH_2*	3.07×10^{-13}
**R3**	$CH_3CH_2\bullet + * \rightarrow CH_2CH_2(g) + H*$	5.92×10^{-1}	*	9.98×10^{-1}
**R4**	$CH_3CH_2* \rightarrow CH_2CH_2(g) + H*$	2.14×10^{-11}		
**R5**	$2H* \rightarrow H_2(g) + 2*$	5.92×10^{-1}		

Table S9. Theoretical rates and surface coverage results calculated from the coverage-dependent kinetic modelling at 873 K over SAPO-34.

No.	Elementary steps	Rate (s^{-1})	Adsorbate	Coverage (ML)
**R1**	$CH_3CH_3(g) + * \rightarrow CH_3CH_2\bullet + H*$	3.91×10^{-4}	H*	2.19×10^{-1}
**R2**	$CH_3CH_2\bullet + * \rightarrow CH_3CH_2*$	-3.81×10^{-3}	CH_3CH_2*	8.04×10^{-8}
**R3**	$CH_3CH_2\bullet + * \rightarrow CH_2CH_2(g) + H*$	3.57×10^{-3}	*	7.80×10^{-1}
**R4**	$CH_3CH_2* \rightarrow CH_2CH_2(g) + H*$	-3.81×10^{-3}		
**R5**	$2H* \rightarrow H_2(g) + 2*$	3.91×10^{-4}		

Table S10. Theoretical rates and surface coverage results calculated from different corrections of adsorbate-adsorbate interactions from 800 to 950 K over Fe/SAPO-34.

	Coverage (ML)				
Temperature	H	Free site	Rate-limiting barrier	TOF(s^{-1})	Adsorbate
800	0.502	0.498	2.34(R1)	6.61×10^{-3}	Coverage-dependent
825	0.500	0.500	2.38(R1)	8.00×10^{-3}	Coverage-dependent
850	0.497	0.503	2.42(R1)	1.40×10^{-2}	Coverage-dependent
875	0.493	0.507	2.46(R1)	2.50×10^{-2}	Coverage-dependent
900	0.491	0.509	2.50(R1)	3.88×10^{-2}	Coverage-dependent
925	0.488	0.512	2.54(R1)	5.47×10^{-2}	Coverage-dependent
950	0.486	0.514	2.58(R1)	7.90×10^{-2}	Coverage-dependent
800	>0.999	<0.001	4.25(R5)	9.69×10^{-10}	Coverage-independent
950	>0.999	<0.001	4.24(R5)	2.10×10^{-5}	Coverage-independent

Table S11. Parameters of the linear curves in Figure 4a for Fe/SAPO-34 and Figure S6 for SAPO-34.

Catalysts	CH_3CH_2-line		CH_2CH_2-line		CH_3CH_2-pressure	CH_2CH_2-rate
	slope	intercept	slope	intercept		
**Fe/SAPO-34**	1.81	0.24	1.81	-0.21	$\mathbf{1.32\times10^{-9}}$	1.64×10^{-2}
**SAPO-34**	1.83	1.87	1.83	-1.87	$\mathbf{1.02\times10^{-9}}$	3.44×10^{-4}

Table S12. Elementary steps for EDH over Fe/SAPO-34 with free energy corrections ΔE_{cor} at different H-covered coverages, where * represents an active site, and CH_3CH_2• represents a CH_3CH_2 radical, i of H_i refers to the number of adsorbed H* initially on the acid sites, / refers to no additional reaction barriers, and energy changes from the initial states to the final states are displayed in Figure S2.

No.	Elementary steps	E_{cor} / eV		
		H_0-Covered Fe/SAPO-34		
		IS	TS	FS
**R1**	CH_3CH_3(g) + * → CH_3CH_2• + H*	-0.40	0.91	-0.64
**R3**	CH_3CH_2• + * → CH_2CH_2(g) + H*	-0.87	/	-0.66
**R4**	CH_3CH_2* → CH_2CH_2(g) + H*	1.15	1.05	-0.66
		H_1-Covered Fe/SAPO-34		
		IS	TS	FS
**R1**	CH_3CH_3(g) + * → CH_3CH_2• + H*	-0.17	1.35	-0.41
**R3**	CH_3CH_2• + * → CH_2CH_2(g) + H*	-0.64	/	-0.55
**R4**	CH_3CH_2* → CH_2CH_2(g) + H*	1.27	1.28	-0.55
		H_2-Covered Fe/SAPO-34		
		IS	TS	FS
**R1**	CH_3CH_3(g) + * → CH_3CH_2• + H*	0.05	0.00	-0.25
**R3**	CH_3CH_2• + * → CH_2CH_2(g) + H*	-0.41	1.14	-0.39
**R4**	CH_3CH_2* → CH_2CH_2(g) + H*	1.52	1.48	-0.39
**R5**	2H* → H_2(g) + 2*	0.45	0.20	-1.12
		H_3-Covered Fe/SAPO-34		
		IS	TS	FS
**R1**	CH_3CH_3(g) + * → CH_3CH_2• + H*	0.21	0.00	-0.06
**R3**	CH_3CH_2• + * → CH_2CH_2(g) + H*	-0.25	1.97	-0.19
**R5**	2H* → H_2(g) + 2*	0.61	0.19	-0.89
		H_4-Covered Fe/SAPO-34		
		IS	TS	FS
**R5**	2H* → H_2(g) + 2*	0.80	0.53	-0.67

Table S13. Elementary steps for EDH over SAPO-34 with free energy corrections ΔE_{cor} at different H-covered coverages, where * represents an active site, and CH_3CH_2• represents a CH_3CH_2 radical, i of H_i refers to the number of adsorbed H* initially on the acid sites, / refers to no additional reaction barriers, the energy changes from the initial states to the final states are displayed in Figure S3.

No.	Elementary steps	E_{cor} / eV		
		H_0-Covered SAPO-34		
		IS	TS	FS
**R1**	$CH_3CH_3(g)$ + * → CH_3CH_2• + H*	-0.40	0.88	-0.64
**R3**	CH_3CH_2• + * → $CH_2CH_2(g)$ + H*	-0.87	/	-1.46
**R4**	CH_3CH_2* → $CH_2CH_2(g)$ + H*	1.07	0.90	-1.46
		H_1-Covered SAPO-34		
		IS	TS	FS
**R1**	$CH_3CH_3(g)$ + * → CH_3CH_2• + H*	-0.17	/	-0.70
**R3**	CH_3CH_2• + * → $CH_2CH_2(g)$ + H*	0.24	/	-0.77
**R4**	CH_3CH_2* → $CH_2CH_2(g)$ + H*	1.23	0.93	-0.77
		H_2-Covered SAPO-34		
		IS	TS	FS
**R1**	$CH_3CH_3(g)$ + * → CH_3CH_2• + H*	-0.01	/	-0.24
**R3**	CH_3CH_2• + * → $CH_2CH_2(g)$ + H*	0.39	1.30	-0.50
**R4**	CH_3CH_2* → $CH_2CH_2(g)$ + H*	1.43	/	-0.50
**R5**	2H* → $H_2(g)$ + 2*	0.17	0.06	-1.12
		H_3-Covered SAPO-34		
		IS	TS	FS
**R1**	$CH_3CH_3(g)$ + * → CH_3CH_2• + H*	0.22	/	-0.06
**R3**	CH_3CH_2• + * → $CH_2CH_2(g)$ + H*	0.63	1.44	-0.19
**R5**	2H* → $H_2(g)$ + 2*	0.62	/	-0.89
		H_4-Covered SAPO-34		
		IS	TS	FS
**R5**	2H* → $H_2(g)$ + 2*	0.81	/	-0.73

Supporting Notes

Note 1: Thermodynamic Corrections

The standard equations were used to perform the thermodynamic corrections, namely the free energy corrections, considering the zero-point energy (ZPE), thermal energy and entropy.[4,5]

Only vibrational motions were included for any surface species. The ZPE correction is calculated by:

$$E_{ZPE} = \sum_i \frac{hv_i}{2} \qquad \text{(S1)}$$

where h is the Plank's constant, and v_i is the vibrational frequency calculated using the harmonic oscillator approximation. The standard vibrational thermal energy is calculated by:

$$U_{vib}^{o} = RT \sum_i \frac{hv_i/k_B}{e^{hv_i/k_BT}-1} \qquad \text{(S2)}$$

where R is the gas constant, and k_B is the Boltzmann's constant. The standard vibrational entropy is calculated as follows:

$$S_{vib}^{o} = R \sum_i \left[\frac{hv_i/k_BT}{e^{hv_i/k_BT}-1} - \ln\left(1 - e^{-hv_i/k_BT}\right)\right] \qquad \text{(S3)}$$

The free energies for adsorbates were corrected by the following the equation:

$$\Delta E_{cor} = E_{ZPE} + U_{vib}^{o} - TS_{vib}^{o} \qquad \text{(S4)}$$

The standard Gibbs free energy (G^o) are calculated as follows:

$$G^o = E_{total} + \Delta E_{cor} \qquad \text{(S5)}$$

where E_{total} refers to the total energy from DFT calculations. In addition, the thermodynamic corrections for any species in the gas phase were obtained by Gaussian code.[6] Moreover, we employed an approximation that the species in the channel of zeolite without adsorption retains 2/3 of the translational entropy of its ideal gas, which was a common practice in previous work.[7] The free energy corrections ΔE_{cor} are shown in Table S12, S13 above.

Note 2: Experimental TOF estimation

The experimental rate of CH_2CH_2 on FeS-1-EDTA was evaluated using the standard TOF formula. The total active sites in the zeolite with the Fe loading of catalyst (m, 0.2 g of catalyst with Fe loading at 0.8 wt%) can be obtained as:

$$total\ site = \frac{m}{M} \times N_A \times n$$

where *M* and *n* refer to the relative atomic mass of iron and the total active sites in one unit cell from the modelling (n = 4 for FeS-1-EDTA in this work due to the local structure). The TOF at 1s was calculated by gas flowing rate (R) of 2 L g_{cat}^{-1} h^{-1} (30% ethane balanced with Ar) and the conversion (X):

$$TOF_{CH_2CH_2} = \frac{R \times N_A}{V} \times X \times \frac{1}{total\ site}$$

where N_A and V refer to the Avogadro constant and the standard molar volume of a gas.

Note 3: Explanation that the TOF value increases with respect to temperature rise

The first elementary step of the reaction, namely the first step dehydrogenation of ethane, is the step from ethane in the gas phase to CH_3CH_2•. When the temperature increases, a larger entropy effect in the free energy correction (-TS) can be expected on the ethane in the gas phase than that in the transition state due to the larger degree of freedom (entropy) of the gaseous ethane. Therefore, the energy difference between the transition state and initial state increases, leading to the barrier increase as the temperature increase.

The rate increase can be explained from the Arrhenius equation with the temperature increase:

$$k = Ae^{\frac{-E_a}{RT}}$$

The reaction barriers calculated at the temperatures of 800 K and 950 K are $E_{800K} = 2.34\ eV$ and $E_{950K} = 2.58\ eV$, respectively. Substituting them into the equation, we can obtain that the exponential term ($\frac{-E_a}{RT}$) at 800 K (-3.39 × 10) is smaller than that at 950 K (-3.15 × 10), illustrating that when the temperature increases, the TOF will increase.

Supporting References

(1) Guo, C.; Mao, Y.; Yao, Z.; Chen, J.; Hu, P. Examination of the Key Issues in Microkinetics: CO Oxidation on Rh (111). *J. Catal.* **2019**, *379*, 52-59.

(2) Chen, J.; Jia, M.; Lai, Z.; Hu, P.; Wang, H. Ssia: A Sensitivity-Supervised Interlock Algorithm for High-Performance Microkinetic Solving. *J. Chem. Phys.* **2021**, *154*, 024108.

(3) Chen, J.-F.; Mao, Y.; Wang, H.-F.; Hu, P. Reversibility Iteration Method for Understanding Reaction Networks and for Solving Microkinetics in Heterogeneous Catalysis. *ACS Catal.* **2016**, *6*, 7078-7087.

(4) Wang, Z.; Liu, X.; Rooney, D.; Hu, P. Elucidating the Mechanism and Active Site of the Cyclohexanol Dehydrogenation on Copper-Based Catalysts: A Density Functional Theory Study. *Surf. Sci.* **2015**, *640*, 181-189.

(5) Cao, X.-M.; Burch, R.; Hardacre, C.; Hu, P. An Understanding of Chemoselective Hydrogenation on Crotonaldehyde over Pt (111) in the Free Energy Landscape: The Microkinetics Study Based on First-Principles Calculations. *Catal. Today* **2011**, *165*, 71-79.

(6) Arooj, Q.; Wilson, G. J.; Wang, F. Methodologies in Spectral Tuning of Dssc Chromophores through Rational Design and Chemical-Structure Engineering. *Mater.* **2019**, *12*, 4024.

(7) Mao, Y.; Wang, Z.; Wang, H.-F.; Hu, P. Understanding Catalytic Reactions over Zeolites: A Density Functional Theory Study of Selective Catalytic Reduction of NO_x by NH_3 over Cu-SAPO-34. *ACS Catal.* **2016**, *6*, 7882-7891.
